# NF-Y activates genes of metabolic pathways altered in cancer cells

**DOI:** 10.18632/oncotarget.6453

**Published:** 2015-12-03

**Authors:** Paolo Benatti, Maria Luisa Chiaramonte, Mariangela Lorenzo, John A. Hartley, Daniel Hochhauser, Nerina Gnesutta, Roberto Mantovani, Carol Imbriano, Diletta Dolfini

**Affiliations:** ^1^ Dipartimento di Scienze della Vita, Università di Modena e Reggio Emilia, Modena, Italy; ^2^ Dipartimento di Bioscienze, Università degli Studi di Milano, Milano, Italy; ^3^ Cancer Research UK Drug-DNA Interactions Research Group, UCL Cancer Institute, Paul O'Gorman Building, University College London, London, UK

**Keywords:** transcription, cancer metabolism, NF-Y, glycolysis, SOCG pathway

## Abstract

The trimeric transcription factor NF-Y binds to the CCAAT box, an element enriched in promoters of genes overexpressed in tumors. Previous studies on the NF-Y regulome identified the general term *metabolism* as significantly enriched. We dissect here in detail the targeting of metabolic genes by integrating analysis of NF-Y genomic binding and profilings after inactivation of NF-Y subunits in different cell types. NF-Y controls *de novo* biosynthetic pathways of lipids, teaming up with the master SREBPs regulators. It activates glycolytic genes, but, surprisingly, is neutral or represses mitochondrial respiratory genes. NF-Y targets the SOCG (Serine, One Carbon, Glycine) and Glutamine pathways, as well as genes involved in the biosynthesis of polyamines and purines. Specific cancer-driving nodes are generally under NF-Y control. Altogether, these data delineate a coherent strategy to promote expression of metabolic genes fuelling anaerobic energy production and other anabolic pathways commonly altered in cancer cells.

## INTRODUCTION

The CCAAT box is an important element present in promoters and enhancers of eukaryotic genes. It is bound by the evolutionarily conserved NF-Y (also named CBF), a trimer formed by NF-YA, NF-YB and NF-YC. NF-YB and NF-YC have histone-like structures, which, upon hetero-dimerization, present a complex surface for NF-YA association, in turn providing sequence-specific CCAAT recognition [1]. The use of genome-wide assays has generalized the concept previously derived by *in vitro* experiments that NF-Y is the primary CCAAT binding protein [2]. Yeast *S. cerevisiae* produces energy and ethanol through glycolysis and fermentation when grown in medium containing glucose; when challenged with non-fermentable carbon sources, yeast cells switch to oxygen-fueled metabolism, by activation of nuclear genes of the mitochondrial respiratory complexes. All these genes contain a CCAAT sequence in their regulatory UAS (Upstream Activating Sequences) sequences and are dependent upon the NF-Y yeast homologue HAP2/3/4/5 [3].

In mammals, the NF-Y regulome is apparently more complex, but it is becoming intelligible, thanks to converging sets of data: (i) the precise biochemical characterization of the target sequence led to the definition of a DNA-binding matrix with high information content, characterized in hundreds mammalian promoters, highlighting a strong positional bias [4]; (ii) Genome-wide experiments confirmed and further extended these observations to enhancers and other genomic regions [5-11]; (iii) Profiling analysis of genes whose expression is affected by functional inactivation of one of the subunits [11-13] found that in addition to a positive role on transcriptional units, NF-Y is also part of repressive mechanisms of transcription.

A common theme in the analysis of the NF-Y regulome in mammalian cells is the presence of the term *metabolism* at the top of Gene Ontology categorizations; functional dissection of individual promoters of metabolic genes indeed indicated the importance of NF-Y for high level of expression. There has been a renewed interest in the transcriptional control of such genes, since specific metabolic pathways are found altered in cancer cells; a vast array of biochemical, genetic and pharmacological data highlight the importance of the expression levels of single genes for “metabolic reprogramming”, an hallmark of the development and progression of tumors [14-16]. This is well exemplified by the recent computational evaluation of expression levels of genes of the SOCG -Serine One Carbon Glycine- pathway across cancer samples, indicating that collective overexpression of genes is predictive of the increased flux of metabolites observed in tumors [17]. Interestingly, analysis of large sets of expression profilings comparing tumors and normal tissues indicate that the NF-Y matrix is enriched in promoters of genes overexpressed in cancer cells [18]; however, it was not determined whether these genes belong to specific pathways. Finally, compelling genetic experiments have recently established that NF-YC, with TAF12 and RAD54L, is a driver oncogene of choroid plexus carcinomas [19].

For these reasons, we decided to take a closer look at the metabolic pathways influenced by the transcriptional activity of NF-Y. We analyzed available genomic data and performed additional gene expression experiments after inactivation of NF-Y subunits, to rationalize its role in the regulation of metabolic genes. The results point to specific pathways, and within them specific nodes, which are under tight NF-Y control.

## RESULTS AND DISCUSSION

### Experimental strategy

Top rank GO terms of NF-Y-regulated genes include metabolic pathways [11-13]. For this reason, we focused specifically on metabolic genes with the following strategy.

(i) We analyzed the results of Affymetrix gene expression profilings of Hela-S3 cells inactivated of NF-YA by shRNA interference [11] and performed additional profilings in epithelial HCT116 and H322 cells under identical conditions of NF-YA inactivation. Fig. S1 shows the levels of NF-YA, as assessed by Western blot analysis; the complete list of genes up- and down-regulated in HCT116 and H322, considering a threshold of 1.3 fold difference, is in Fig. S2. Moreover, we analyzed the recently reported profilings data of mouse embryonic stem (mES) cells functionally inactivated of individual and of all three subunits of NF-Y [12].

(ii) To validate profilings, we analyzed the expression of selected genes of the affected pathways by qRT-PCR after inactivation of the NF-YB subunit in Hela-S3 cells with two different shRNAs.

(iii) To match function with location, we analyzed ChIP-Seq data of NF-YA and NF-YB in Hela-S3, GM12878 and K562 derived from ENCODE [9, 11], as well as the individual binding of the three subunits in mouse ES cells [12]. Specifically, we verified binding to the *bona fide* CCAAT matrix in the prototypical promoter position, between -60 and -100 from the TSS [4], as well as in distal locations classified as enhancers by virtue of the presence of appropriate epigenetic marks (H3K27ac, H3K4me1).

The lack of NF-Y functional dependence in the presence of promoter binding might be due to an effective CCAAT-independence of the gene -and activity of compensatory TFs- or to the long half-life of the specific mRNA—the latter is a particularly relevant point, as we consider here housekeeping genes involved in basic metabolic functions, whose mRNAs tend to be relatively stable.

### Overall density of NF-Y regulated genes in metabolic pathways

**Figure 1:**
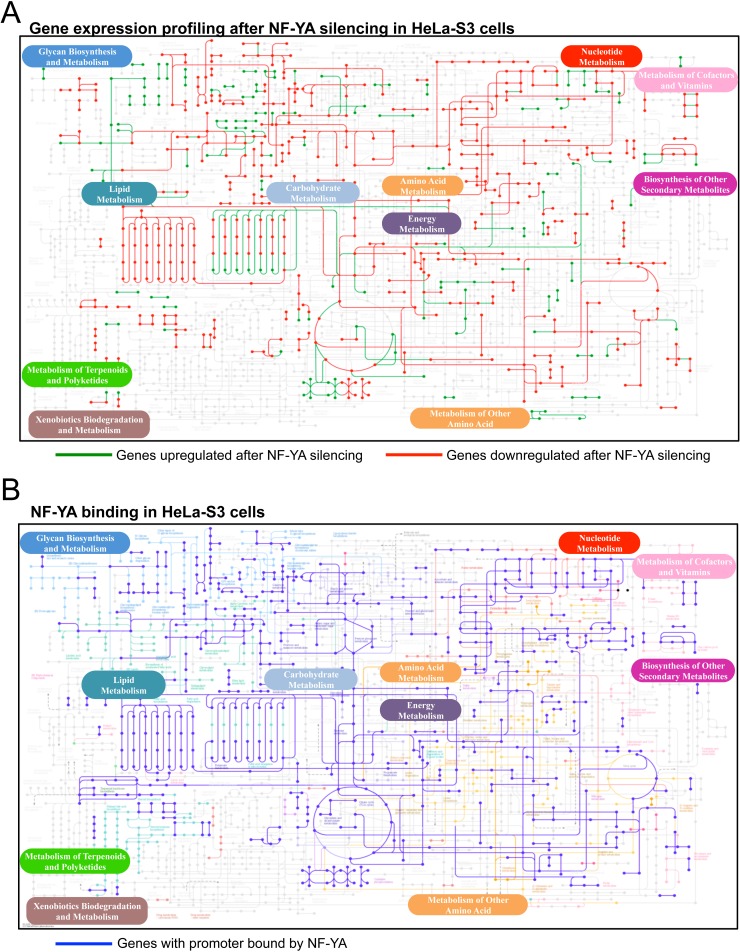
NF-Y targets in metabolic pathways Global map of metabolic pathways targeted by NF-YA. **A**. Metabolic genes upregulated (in green) and downregulated (in red) after silencing of NF-YA in HeLa cells [11]. **B**. Metabolic genes with core promoters bound by NF-YA are indicated in blue according to ENCODE ChIP-Seq data. The maps are constructed with the KEGG Mapper v 2.5 tool.

The profilings and ChIP-Seq data of Hela-S3 cells were first analyzed within the KEGG global metabolic chart. Genes whose expression is decreased after NF-YA inactivation are in red and those increased are in green (Fig. 1A); genes with core promoters bound by NF-YA are in blue (Fig. 1B). The charts of gene expression profilings of human H322 and HCT116 cells after NF-YA, and of mouse ES cells after NF-YA, NF-YB and NF-YC inactivation, are shown in Figs. S3 and S4. Genes bound by NF-YB in Hela-S3, showing marginal differences with NF-YA, are in Fig. S5. Similarly, NF-YA and NF-YB binding in K562 and GM12878, as well as NF-YA, NF-YB and NF-YC in mES cells are shown in Figs. S6-8. A bird's eye view of the results collectively indicates high densities in areas of lipids, carbohydrates and nucleotides metabolisms. Other locations -glycan, vitamins, terpenoids and xenobiotics- are mostly depleted, with only selected genes positive. Aminoacids pathways show density in specific areas. We calculated the statistical significance of the regulated pathways in profilings of Hela-S3 and mES cells using KOBAS 2.0 [20], a software providing a better definition for the different metabolic sub-pathways. This led to the identification of globally enriched terms (Fig. S9). As previously reported [11], the Hela-S3 pathways at the top of the list are related to *cell-cycle* and *mitosis*, but specific metabolic terms are enriched and they are indeed predominant in mES cells. Focusing specifically on metabolic terms (Table 1), we note that anabolic genes are less expressed after NF-Y removal, whereas catabolic ones tend to be increased; among the formers, *aminoacids* -notably Ala, Asp, Glu; Ser, Gly; Gln- *lipids* and *nucleic acids* are enriched. As for carbohydrate, *carbon metabolism* -mostly glycolysis- is enriched. We hereafter further examine the role of NF-Y in such pathways.

**Table 1:** Metabolic terms enriched in genes whose expression is changed upon inactivation of NF-YA in Hela-S3 cells (Left panel) and in mouse ES cells (mESC) (Right panel). Pathways and Gene Ontology analyses were performed with Kobas 2.0 and metabolic terms extracted from the list. The full list of terms enriched in differentially expressed genes is shown in Fig. S9

HeLa-S3				mESC			
	*CATEGORY*	*ID*	*P-Value*	*CATEGORY*	*ID*	*P-Value*	
**DOWNREGULATED**	Alpha-amino acid biosynthetic process	GO:1901607	3.73E-04	Carboxylic acid metabolic process	GO:0019752	7.86E-16	**DOWNREGULATED**
	Cellular amino acid biosynthetic process	GO:0008652	7.16E-04	Metabolism	REACT_188937	3.20E-13	
	Alanine, aspartate and glutamate metabolism	hsa00250	7.93E-04	Small molecule metabolic process	GO:0044281	2.51E-12	
	Serine glycine biosynthesis	P02776	1.48E-03	Metabolic pathways	mmu01100	5.73E-11	
	Glutamine metabolic process	GO:0006541	2.86E-03	Cellular amino acid metabolic process	GO:0006520	4.98E-10	
	Amino acid synthesis and interconversion (transamination)	REACT_238	3.39E-03	Small molecule biosynthetic process	GO:0044283	6.69E-10	
	Metabolism of polyamines	REACT_14820	3.39E-03	Carbon metabolism	mmu01200	8.45E-08	
	Glutamine family amino acid metabolic process	GO:0009064	4.02E-03	Oxidation-reduction process	GO:0055114	1.65E-07	
	Polyamine metabolic process	GO:0006595	4.48E-03	Biosynthesis of amino acids	mmu01230	2.76E-07	
	Glycemic traits (pregnancy)	NHGRI GWAS	4.49E-03	Metabolism of amino acids and derivatives	REACT_232845	1.63E-06	
	L-serine metabolic process	GO:0006563	7.32E-03	Amino acid synthesis and interconversion (transamination)	REACT_239550	4.43E-06	
	Purine nucleobase biosynthetic process	GO:0009113	1.01E-02	Glycolysis / Gluconeogenesis	mmu00010	6.44E-06	
	Nucleotide catabolic process	GO:0009166	1.05E-02	Glycine, serine and threonine metabolism	mmu00260	1.87E-05	
	Fatty acid biosynthesis	hsa00061	1.94E-02	Glucose metabolism	REACT_261601	3.03E-05	
	Purine metabolism	hsa00230	2.92E-02	Fatty acid biosynthetic process	GO:0006633	3.70E-05	
				Cholesterol metabolic process	GO:0008203	6.50E-05	
**UPREGULATED**	Cellular catabolic process	GO:0044248	2.24E-06	Lipid metabolic process	GO:0006629	6.74E-05	
	Carboxylic acid catabolic process	GO:0046395	7.94E-05	Fructose and mannose metabolism	mmu00051	8.88E-05	
	Fatty acid catabolic process	GO:0009062	1.03E-04	Pyruvate metabolic process	GO:0006090	9.33E-05	
	Lipid oxidation	GO:003440	1.14E-04	Valine, leucine and isoleucine degradation	mmu00280	1.49E-04	
	Autophagy	GO:0006914	1.84E-04	Aspartate family amino acid metabolic process	GO:0009066	3.12E-04	
	Monocarboxylic acid catabolic process	GO:0072329	2.42E-04	Glycogen storage diseases	REACT_203949	4.37E-04	
	Branched-chain amino acid catabolism	REACT_197	1.12E-03	Metabolism of carbohydrates	REACT_248571	4.37E-04	
	Fatty acid beta-oxidation	GO:0006635	1.17E-03	Arginine and proline metabolism	mmu00330	6.05E-04	
	Macroautophagy	GO:0016236	1.93E-03	Activation of gene expression by SREBF (SREBP)	REACT_198969	8.19E-04	
	Protein catabolic process	GO:0030163	2.60E-03				
	Valine, leucine and isoleucine degradation	hsa00280	2.73E-03	Regulation of cellular macromolecule biosynthetic process	GO:2000112	1.10E-05	**UPREGULATED**
	Macromolecule catabolic process	GO:0009057	2.84E-03	Regulation of cellular biosynthetic process	GO:0010556	3.43E-05	
	Cellular response to starvation	GO:0009267	3.01E-03	Regulation of macromolecule metabolic process	GO:0006355	4.52E-05	
	Ribonucleoside bisphosphate metabolic process	GO:0033875	7.23E-03	Regulation of biosynthetic process	mmu05220	5.70E-05	
	Purine nucleoside bisphosphate metabolic process	GO:0034032	7.23E-03	modification-dependent macromolecule catabolic process	mmu04068	1.59E-04	
	Nucleotide metabolic process	GO:0009117	7.31E-03	MyD88:Mal cascade initiated on plasma membrane	GO:0031326	1.69E-04	
	Aerobic respiration	GO:0009060	1.64E-02	Toll Like Receptor TLR6:TLR2 Cascade	GO:0016070	1.72E-04	
	Nucleotide-sugar metabolic process	GO:0009225	1.67E-02	Ubiquitin-dependent protein catabolic process	GO:0060255	2.14E-04	
	Glycosphingolipid metabolism	REACT_116105	1.68E-02	Modification-dependent protein catabolic process	GO:0009889	2.25E-04	
	Metabolism of lipids and lipoproteins	REACT_22258	2.15E-02	Clathrin derived vesicle budding	GO:1903507	3.17E-04	
	Citrate cycle (TCA cycle)	hsa00020	5.24E-02	Negative regulation of macromolecule metabolic process	REACT_206529	3.33E-04	
	Fatty acid, triacylglycerol, and ketone body metabolism	REACT_22279	6.20E-02	Regulation of lipid metabolism by PPARalpha	REACT_263004	3.58E-04	
	Cytochrome c-mediated apoptotic response	REACT_831	6.91E-02	Signaling by PDGF	GO:0006511	3.78E-04	
	Oxidative phosphorylation	hsa00190	7.55E-02	Hippo signaling pathway	REACT_198602	7.65E-04	

### Lipids metabolism

**Figure 2:**
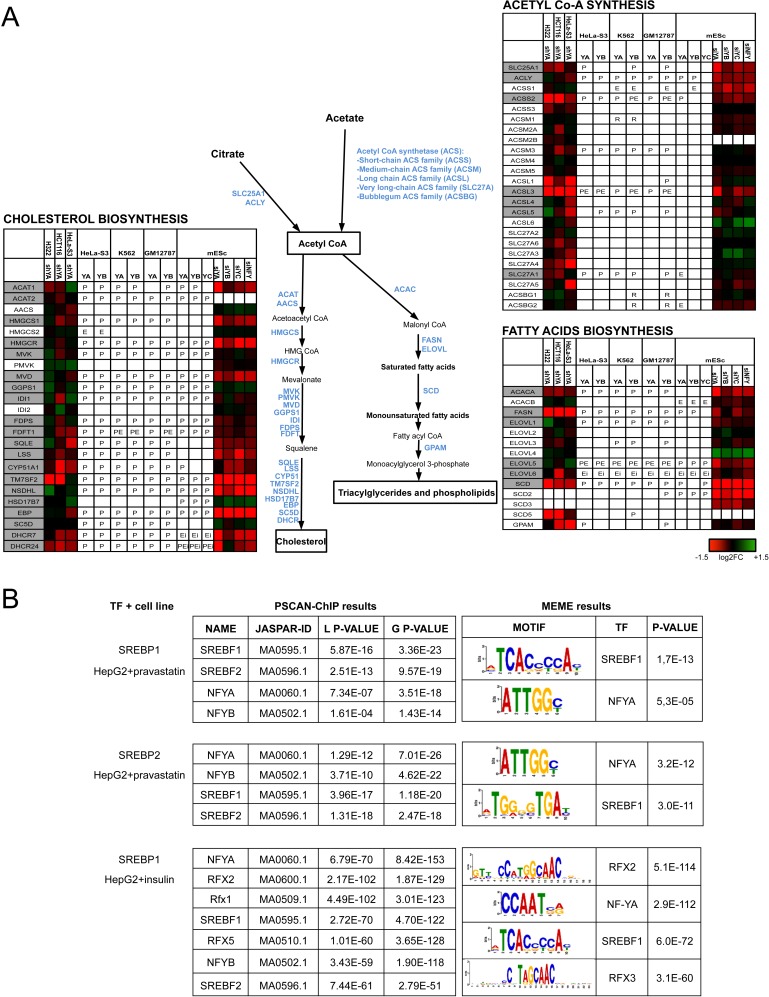
NF-Y activates genes of lipid metabolism **A**. Genes involved in cholesterol and fatty acids metabolisms are shown. The heatmap represents the log2 fold change of relative expression derived from profiling analysis (See colour scale) in the indicated cell lines after inactivation of NF-Y subunits: H322 (This manuscript, Fig. S2), HCT116 (This manuscript, Fig. S2), HeLaS3 [11] and mESC [12]. The presence of NF-Y binding in ENCODE datasets is indicated with P (core promoter binding), E (external enhancer), Ei (enhancer in gene body) or R (repetitive sequence within 5 kb from the TSS). *In vivo* binding of SREB-1/2 according to ChIP-Seq experiments is indicated by a grey background. **B**. Analyses of over-represented motifs in SREBPs peaks in HepG2 ChIP-Seq data analyzed by ENCODE. The matrices were derived with Pscan-ChIP (Left panel) and MEME (Right panel), and the relative p-values are shown.

NF-Y activates genes of the two main branches of lipids metabolism, cholesterol and fatty acids (Fig. 2A): inactivation leads to decreased expression of most cholesterol genes, with the exception of HMGCS2, GGPS1, IDI1/2, HSD17B7, SC5D (Fig. 2A). A similar situation is observed for the fatty acids branch. With the exception of HMGCS2, IDI2 and PMVK, all promoters are bound *in vivo* in most cell types, including those negatively regulated in mES cells. Rate limiting enzymes of cholesterol -HMGCS1, HMGCR, Squalene Synthase (SQLE), DHCR - and fatty acids pathways -Acetyl COA Carboxylase (ACACA), Fatty Acids Synthase (FASN), Steatoryl CoA Desaturase (SCD)- were previously reported to be under NF-Y positive control [21-36]. Acyl-CoA lyase (ACLY) and Acetyl-CoA synthase of the short chains (ACSS2) are also controlled.

Sustained proliferation rates of cells require a high level of *de novo* biosynthesis of lipids, which are incorporated into newly formed cell membranes. Indeed, these pathways are crucial for cancer cells, and many genes are overexpressed [37]. What is particularly relevant is that the genes more sensitive to NF-Y-inactivation are also those implicated in cancer development. On the cholesterol side, the mevalonate genes are overexpressed in many cancers, including HMGCS1, indeed a cancer-driving gene [38], and the target of statins, a promising “new” class of anti-cancer drugs [39]. In the fatty acids branch, NF-Y targets FASN, SCD1 and GPAM, frequently overexpressed in tumors; FASN is a driver of neoplastic transformation [40]. ACSS2 is essential for transformation of acetate to Acetyl-CoA, thereafter used for lipids synthesis, as well as for histone acetylations [41,42]; expression of ACSS2 is high in many types of tumors and KO mice are protected in mouse models of liver carcinogenesis. The parallel strong NF-Y positivity of the less studied ACSM3 and ACSL3 invites evaluation of their expression levels across cancer samples, and role in tumorigenesis. Finally, ACLY is at the crossroad of lipids and glucose metabolism, mediating the production of Acetyl-CoA by transformation of cytoplasmic citrate to oxaloacetate. ACLY deregulation is generally observed in cancer, often correlating with tumor stage and prognosis [43] and silencing blocks proliferation of cancer cells by different mechanisms, impairing lipid biosynthesis, glycolytic enzymes, citrate accumulation and histones acetylation.

SREBP-1 and SREBP-2 are the master TFs of cholesterol and fatty acids biosynthesis [44]; the SREBP-1 gene itself is controlled by NF-Y [45, 46]. Genome-wide experiments reported a significant overlap between NF-YA and SREBP-1 sites [7], and enrichment of NF-Y sites in SREBP-1 peaks in mouse liver cells [47]. ChIP-Seq data of SREBP-1/2 in HepG2 and GM12878 were recently reported by the ENCODE consortium: inspection of binding to lipidogenic genes indicates a perfect correlation with NF-Y in cholesterol genes, and near perfect in fatty acids genes (SREBP1/2 positive promoters are marked in grey in Fig. 2A). We thus decided to analyze the global genomic overlap of NF-Y and SREBP-1/2: to do so, we used the PScan-ChIP software [48], which allows the identification of Transcription Factor Binding Site (TFBS) present in the JASPAR or TRANSFAC databases, as enriched in close proximity -<75 bps- of genomic peaks of a given TF. The statistical enrichment of positively correlating TFBSs is measured as “global” or “local“, depending on the robustness of the overlap (Fig. S10). The results of such analysis for SREBP-1 and SREBP-2 are shown in Fig. 2B: the NF-Y consensus present in JASPAR -termed NFYA or NFYB- is significantly enriched in SREBP-1 and SREBP-2 peaks in HepG2 cells treated with pravastatin, but not in untreated GM12878 (Fig. S10); similarly, SREBP-1 peaks are enriched in CCAAT boxes in HepG2 cells treated with insulin. For SREBPs in pravastatin treated HepG2, the program signals “global” enrichments -p values of 10^−20^- indicating that the CCAAT box is a “primary” binding site. By comparison, the SREBF motif has similar p values, indicating that the majority of SREBP-1 genomic sites in HepG2 have both a canonical SRE and a CCAAT box. No other TFBS is significantly enriched. The data of SREBP-1 peaks of insulin-treated HepG2 are very similar, except that RFX motifs also emerge, with similar frequencies (Fig. 2B). To validate these results, we used the MEME tools for *de novo* motif discovery, to inspect SREBP-1/2 genomic peaks: we indeed retrieved logos identical to the NF-Y binding site -the ATTGG reverse of CCAAT- in addition to the expected SRE sequence, and, in insulin-treated HepG2, the RFX logo (Fig. 2B). Thus, two types of analysis indicate that NF-Y and SREBPs share a vast set of genomic locations, extending beyond lipidogenic genes, at least in liver cells. Intriguingly, this is not observed in untreated GM12878 B cells, possibly suggesting treatment- or cell-type specific regulation.

Mechanistically, NF-Y and SREBPs are believed to serve different functions in transcriptional activation: the former as a “pioneer” TF, which binds to promoters and predispose a positive chromatin environment [11, 12, 49, 50]; SREBPs as “activator” TFs promoting transcriptional elongation. In fact, nuclear translocation and DNA-binding of SREBPs are visible under specific conditions: typically, this requires relocation from the ER into the nucleus, following addition of PTMs and proteolysis, according to specific stimuli [44, 47].

### Respiratory genes, glycolysis and the TCA cycle

**Figure 3:**
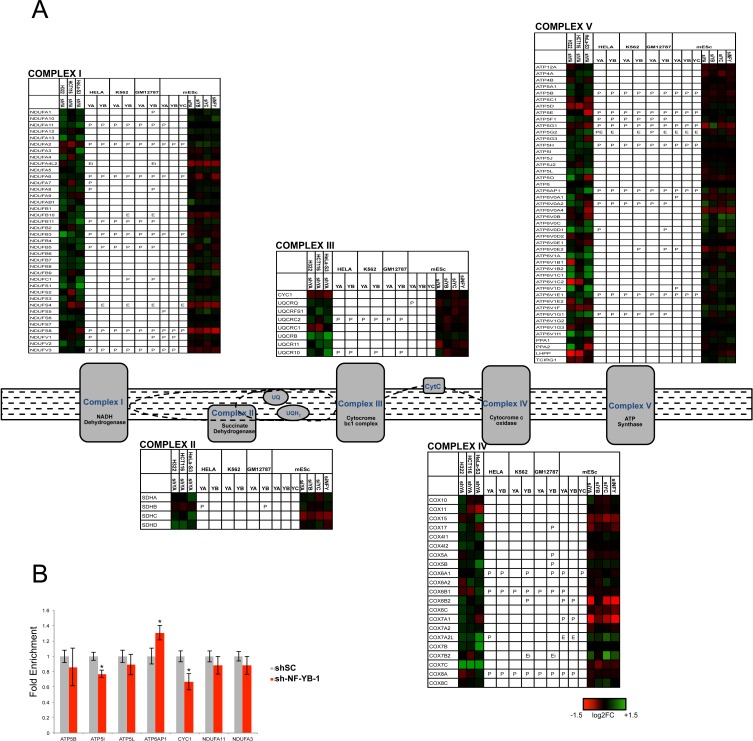
NF-Y and expression of respiratory genes **A**. Genes of oxidative phosphorylation chain complexes are shown with the relative expression levels after inactivation of NF-Y in different cell lines, and the relative presence of NF-Y binding in P (promoter), E (external enhancer), Ei (enhancer in gene body) or R (repetitive sequence within 5 kb from TSS). **B**. qRT-PCR evaluation of expression levels of respiratory genes after inactivation of NF-YB in Hela cells. The average −/+ SD of three biological replicates is represented. (*p<0.05).

The NF-Y homologue HAP2/3/5 is the master transcriptional activator of all genes of the respiratory chain in yeast, following the shift from fermentable (glucose) to non-fermentable (lactate) carbon sources [3]. The promoters of two human respiratory chain genes -ATP Synthase β/ATP5B and CYC1- were originally shown to rely on CCAAT boxes [51-54], and it could be inferred that NF-Y globally controls mammalian respiratory genes as well. The mitochondrial electron transport chain is composed of five complexes (I-V), each containing multiple subunits encoded by nuclear and mitochondrial genes. Surprisingly, we notice differential densities, both in genomic binding and transcriptional activation, in the areas of oxygen-fueled (low) and carbohydrate (high) metabolisms (Fig. 1); this is matched by KOBAS analysis, with terms such as *aerobic respiration* and *oxidative phosphorylation* being up-regulated upon NF-YA inactivation, hence normally repressed (Table 1). Further analysis of the genes of the 5 respiratory complexes indicates the following: CYC1 (complex III) is -modestly- down-regulated in NF-Y-inactivated cells, but not bound by NF-Y; ATP5B (complex V) is bound and regulated; in general, however, only a handful genes -NDUFA2, NDUFS8, ATP5E, ATP5G1 and SDHB- are directly activated in cancer cells, eight genes in mES cells (Fig. 3A). A larger set of genes is repressed by NF-Y, either through direct binding, or indirectly. We verified the expression analysis by inactivation of NF-YB through shRNA interference of Hela cells: of seven representative genes analyzed by qRT-PCR, either bound or not by NF-Y *in vivo*, four showed negligible changes, ATP6AP was modestly increased and CYC1 was modestly decreased, in accordance with the profilings data (Fig. 3B). Experiments using a second shRNA directed against NF-YB yielded similar results (Fig. S11). In summary, NF-Y activation of respiratory genes is globally an exception, and expression of most genes are either not controlled, or repressed.

**Figure 4:**
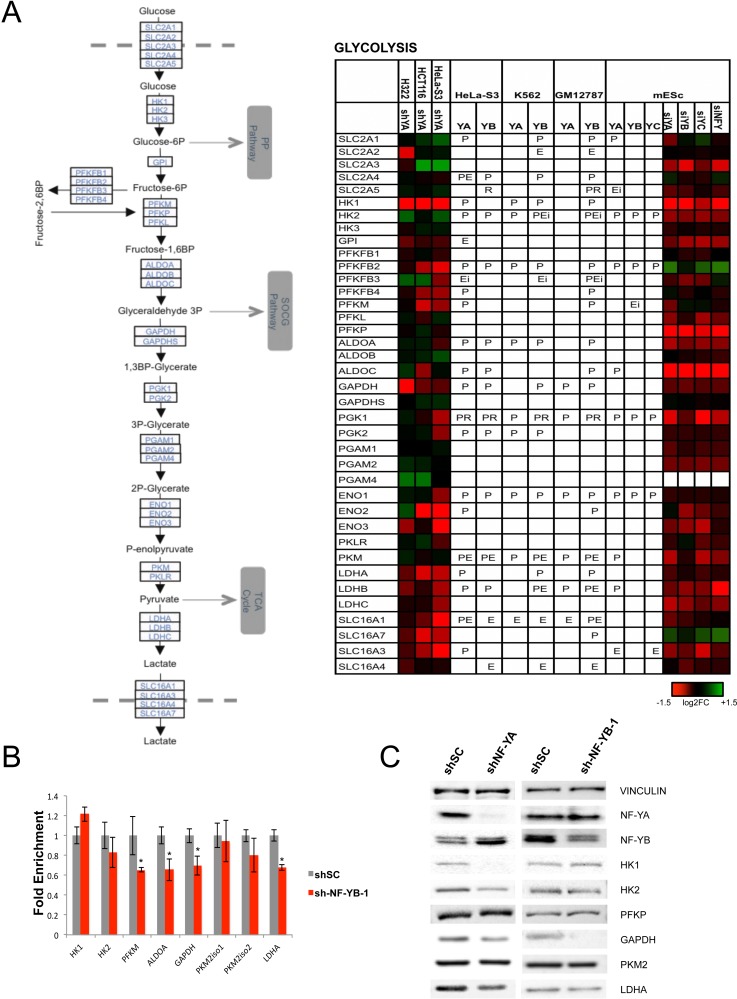
Glycolytic enzymes are regulated by NF-Y **A**. Genes encoding for glycolytic enzymes are shown with their expression levels after inactivation of NF-Y in different cell lines, and the presence of NF-Y binding is indicated as in Fig. 3. **B**. qRT-PCR evaluation of expression levels of selected genes after inactivation of NF-YB in Hela cells. The average −/+ SD of three biological replicates is represented. (*p<0.05). **C**. Western blot analysis of protein levels of NF-Y targets in Hela cells inactivated of NF-YA (Left panel), or NF-YB (Right panel).

On the other hand, glycolytic genes are densely populated of NF-Y-activated units (Figs. 1 and 4A). The sensitivity of such genes to NF-Y inactivation is very high in mES cells; PFKFB2 and SLC16A7 are the only genes showing up-regulation. Critical genes such as PFKFB, PGK, GAPDH, PKM, LDH are bound and heavily rely on NF-Y activity. Note that Aldolase B, one of the first NF-Y promoters dissected [55, 56], is negative, possibly because it is mostly expressed in liver cells, not assayed here. Regulation is similar in cancer cells, with differences in SLC2A3, SLC16A7, PFKFB2/3 (in HCT116 and H322), PKM and HKII. Note that HKII and PKM promoters were shown to be NF-Y-dependent [57-59]. We verified the expression of some of these genes in NF-YB-inactivated Hela-S3 cells by qRT-PCR: most are decreased, with the exception of HKI, HKII and PKM (Fig. 4B and Fig. S11). We also checked the protein levels of several glycolytic enzymes, and found HKI, HKII, GAPDH and LDHA to be decreased, whereas PFKP was unchanged (Fig. 4C). These data are in agreement with the mRNA analysis, with two exceptions: HKII, whose protein is decreased and mRNA is increased after NF-YA inactivation, and PKM, showing no change at the protein level. The differential effects could be due to the long half-life of the proteins, which would require assessment at longer times after NF-Y removal, or to the previously reported differences in sets of genes regulated by NF-YA and NF-YB inactivation [13].

Downstream of pyruvate, Acetyl-CoA enters the TCA cycle, which is functionally linked to mitochondrial oxidative phosphorylation: at the level of binding, the density of NF-Y sites in promoters is high, but the regulation is apparently complex and, intriguingly, somewhat dissimilar in cancer and mES cells (Fig. S12A). The Pyruvate Dehydrogenase -PDH- complex, leading to Acetyl-CoA production by oxidation of pyruvate, illustrates such complexity: PDHA1 is affected, but not the DLD and DLAT subunits, which are, if anything, repressed by NF-Y; at the same time, the regulatory PD Kinase -PDK1/4- which inhibits the activity of the PDH complex, is robustly activated by NF-Y, as shown before [60]. Parallel pathways feed into oxalacetate from: (i) Phosphoenol-pyruvate *via* PCK (PEPCK), positively controlled by NF-Y, notably PCK2; (ii) Pyruvate Carboxylase (PC), which is bound -promoter and enhancer- and negatively regulated (Fig. S12A). The control of PC by NF-Y is also complex [61], including positive effects upon removal of single repressive CCAAT boxes [62]. Note the PC route is crucial for anaplerosis, that is, replenishment of the pools of the TCA cycle intermediates [63]. Production of citrate from Acetyl-CoA and oxaloacetate, and further to isocitrate, is mediated by Citrate synthase and Aconitase, whose promoters are bound *in vivo*, but modestly affected. Thereafter, enzymes are not uniformly regulated: Succinate Dehydrogenases (SDH) and Malate Dehydrogenase (MDH) are repressed by NF-Y in cancer cells (Fig. S12A). For IDH1/2, OGDH and FH, there is less significant regulation in the different cancer cell types analyzed, and a positive, indirect, effect on IDH2 in mES cells. We assayed some of the TCA genes by qRT-PCR after NF-YB inactivation in Hela cells: consistent with the profilings, PDHB, PDK1, ACLY but not PDHA1, are decreased, whereas OGDH, IDH2 and DLD are unchanged (Fig. S12B). In the second set of experiments using shNF-YB-2, the data are similar, except that OGDH is decreased, and PDHB is not, as in the profilings, (Fig. S11). The protein levels of PDHA were evaluated in Western Blots and found to be unchanged (Fig. S12C). Note that NF-Y appears to promote expression of enzymes converting α-ketoglutarate and malate out of the TCA cycle (See below). In summary, NF-Y does bind to most promoters of TCA cycle genes, but its role is less relevant, and possibly cell-type specific; in general, it does not uniformly favor the progression of Acetyl-CoA derived from glucose, lipids and aminoacids catabolism through the oxidative production of energy, particularly in cancer cells. Again, this is in striking contrast to the results obtained in yeast, where the CIT1, IDH, FH and MH genes are robustly activated by the HAP2/3/4/5 complex during activation of the respiratory response [64].

A major and unexpected finding of our analysis is that the control of energy metabolism by NF-Y changed during evolution, from promoting mitochondrial energy production, in *fungi*, to activating genes of anaerobic pathways, in mammals. The preference for anaerobic energy production by glycolysis in the presence of oxygen is a hallmark of cancer cells, a phenomenon known as the Warburg effect [65]. This requires a high activity of glycolytic enzymes; accordingly, the overall levels of many of them are elevated in many types of tumors, with respect to normal cells, mainly because of increased mRNA expression [66].

In general, NF-Y appears to have a coherent strategy of promoting glucose, but not oxygen utilization. Among glycolytic enzymes, GAPDH produces Glyceraldeyde-3P, which enters the biosynthetic pathway of Serine biosynthesis (See below). PKM promotes the final, rate-limiting step of glycolysis to generate pyruvate and ATP and is important in cancer cells [67]. Lactate Dehydrogenases are key to drive regeneration of NAD^+^ and continuous ATP production from glycolysis. Lactate Dehydrogenase is produced by two genes -LDHA and LDHB- whose relative expression varies depending upon the cellular status: in cancer cells, it is shifted toward LDHA, generating the LDH5 enzyme, most efficient in driving the Pyruvate-to-Lactate conversion required to keep the glycolytic flux flowing [68]. It is thus noteworthy that NF-Y is more important for LDHA expression in cancer cells, and for LDHB in mESCs. At the end of glycolysis, NF-Y does not appear to promote a flux of Acetyl-CoA entering the TCA cycle: this is exemplified by the modest impact on PDH, on the associated subunits, and activation of its repressor PDK. At the same time, NF-Y promotes exit of α-ketoglutarate and malate from the TCA cycle to enter alternate pathways, in cancer cells at least (See below).

Long time considered as housekeeping genes subject to modest levels of regulation, the promoters of these genes recently regained interest, but information about the DNA elements and TFs acting on many of them remains scarcely abundant. HIF-1α, MYC, STAT3 and p53 are TFs associated with the regulation of glycolytic enzymes [69-74]. The formers are generally activators, while p53 is inhibitory, at least in the wt configuration. HIF-1α is activated by anaerobic stress and constitutively active in many cancer cells, targeting essentially all glycolytic genes; MYC was shown to interact with NF-Y directly [75] and the peaks of MYC and NF-Y overlap significantly in ENCODE data [11]. Interestingly, p53 was shown to promote gluconeogenesis [76], while oncogenic Gain-of-Function mutations of p53, reported to impact on expression of NF-Y genes [77, 78], mediate metabolic reprogramming and increased glycolysis [79]: it will be interesting to evaluate the effects of mutp53 on the metabolic genes targeted by NF-Y identified here.

### The SOCG and Glutamine pathways

**Figure 5:**
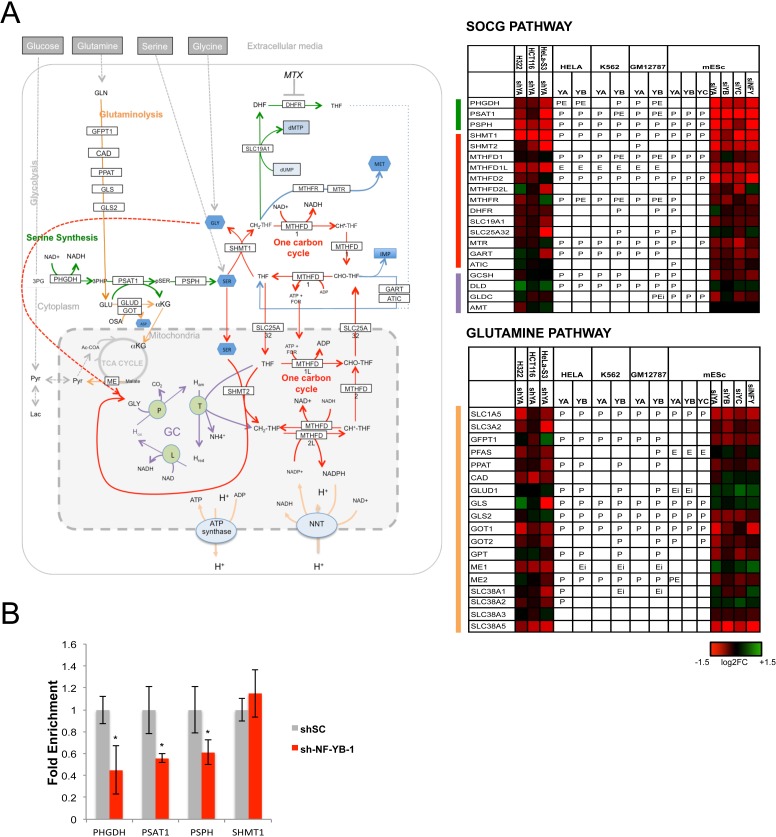
Activation of SOCG genes by NF-Y **A**. Genes of the SOCG (Serine, One Carbon, Glycine) pathway are shown with the expression levels after inactivation of NF-Y, and the presence of NF-Y binding as in Fig. 3. **B**. qRT-PCR evaluation of expression levels of PHGDH, PSAT1, PSPH, SHMT1 after inactivation of NF-YB in Hela cells. The average −/+ SD of three biological replicates is represented (*p<0.05).

We noticed high densities of NF-Y targets in specific pathways of Serine/Glycine, Glutamine and Methionine, all enriched in KOBAS analysis (Table 1). The SOCG pathway is central for *de novo* production of Serine and Glycine, and for additional metabolisms -folates, nucleotides- crucially required for growing cells [80]. NF-Y binding is present on all promoters of the pathway in at least one cell type with the exception of the SLC19A1 transporter, MTHFD2L and AMT (Fig. 5A). Serine synthesis stems from a branch of glycolysis, through Glyceraldeyde 3P being sequentially metabolized by three enzymes: PHGDH, PSAT and PSHP; the promoters are all bound, and the mRNAs decreased upon NF-YA removal. For the PHGDH promoter, genetic evidence of the importance of the CCAAT box was reported [81]. Conversion to Glycine is then obtained through the activity of SHMT, present in two isoforms, one located in the cytoplasm -SHMT1- the other -SHMT2- in mitochondria: NF-YA regulates the former, but apparently not the latter. qRT-PCR analysis of Hela-S3 cells inactivated with the two shRNAs against NF-YB confirmed the control of PHGDH, PSAT1, PSPH, whereas SHMT1 was, if anything, positively affected (Fig. 5B and Fig. S11). Interestingly, NF-Y also impacts on the mitochondrial branch, by activation of two key enzymes, MTHFDL1 and MTHFD2. Control of MTHFR and DHFR signals a further role on folates metabolism, which is linked to Methionine synthesis and nucleotides production (See below). In summary, the data indicate that NF-Y plays a crucial role in the activation of SOCG genes.

Glutamine is another pathway dense with NF-Y targets, at the level of promoter recognition and function (Fig. 5A). Binding of NF-Y is absent only in SLC3A2, CAD, SLC38A5 and SLC38A3; the latter gene, GLUD1 and GPT are unchanged in expression after NF-YA-inactivation in cancer cells. Most genes are activated by NF-Y through direct promoter binding [82]. Specifically, GLS and GLS2 are differentially regulated in Hela-S3 and mES cells: the former is bound and activated in Hela-S3 (but repressed in H322), but not regulated in mES cells. The reciprocal is true for GLS2. These enzymes are crucial for the conversion of Glutamine to Glutamate, which can then be transformed to α-ketoglutarate by GLUD1, not regulated by NF-Y in cancer cells and modestly repressed in mES cells. This branch is a second leg of anaplerotic mechanisms filling the TCA cycle with metabolites from the Glutamine pathway: the positivity of GOT1/2, but not GLUD1, is yet another indication that NF-Y is not favoring anaplerosis *per se*. GOT1/2, in fact, convert Aspartate from Glutamine into oxaloacetate, then converted into malate [83]: thereafter, the Malic enzymes ME1 ME2, both regulated by NF-Y (Fig. 5A), mediate pyruvate production and exit from TCA. In summary, NF-Y appears to promote a shift of Glutamine catabolism from the TCA cycle into alternative biosynthetic pathways, matching the modest role exerted on PDH (Fig. S12A). The negative regulation of SDH (Fig. 3A and S12A) and the activation of genes deviating metabolites from the TCA cycle are a further indication that NF-Y is not promoting oxygen-mediated production of energy.

The Glutamine and SOCG pathways are altered in cancer cells. As to the former, tumors usually become addicted to high levels of Glutamine. The key GLS2 is activated by p53 [84, 85], and regulation is shared by p63 and p73, with a different outcome depending from the isoforms [86, 87]. Genes of the SOCG pathways are overexpressed in cancer [88], the global alteration directly impinges on an altered flux of metabolites [17]. PHGDH and SHMT2 are predictive of survival outcomes in breast cancer, PSPH of hepatocellular carcinomas [89, 90]. SHMT2 is a cancer driver gene [91, 92]; PHGDH overexpression, resulting from genomic amplification, is essential for growth of certain human breast cancers [93] and melanomas [94]; PSAT1 is overexpressed in colon cancer [95]. In general, there are very few data on the mechanisms of transcriptional regulation of SOCG pathway genes: p53 is a repressor of PHGDH [96], whereas cMYC overexpression leads to increased biosynthesis of serine [90]. The identification of NF-Y/CCAAT as a pivotal element should facilitate the identification of neighboring elements and TFs involved in regulation of these units.

### Polyamine and purine metabolism

**Figure 6:**
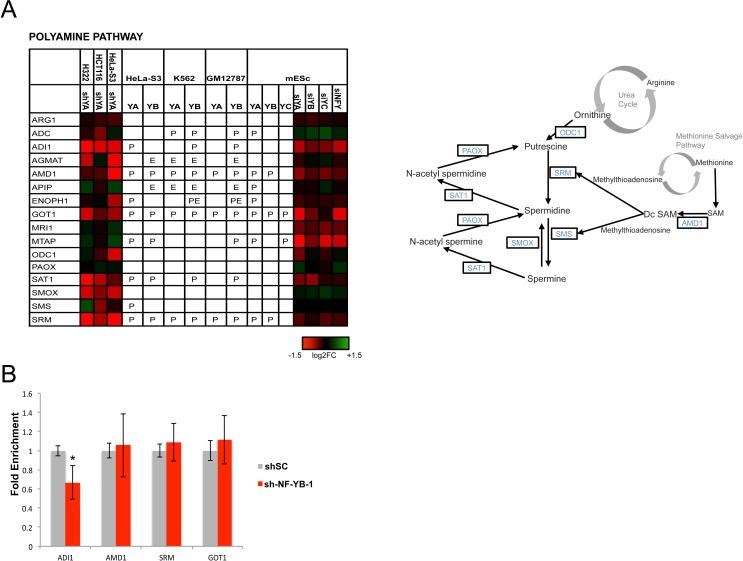
Activation of genes of the polyamine metabolism by NF-Y Genes of the polyamine pathway are shown with the expression levels after inactivation of NF-Y, and the presence of *in vivo* NF-Y binding as in Figs. 3-5.

The biosynthetic pathways of purines and polyamines have high density of NF-Y-controlled genes (Fig. 1 and Table 1). In the polyamine pathway, which is extremely important for cancer cells (97), there are two rate-limiting steps, mediated by ODC1, from the Urea cycle, and AMD1, from the Methionine salvage pathway. AMD1 is directly controlled by NF-Y in cancer and mES cells, but NF-Y inactivation also leads to an indirect decrease of ODC1 (Fig. 6A). Additional regulated genes are ADI1, AGMAT, GOT1, SMOX, SMS and SRM. ADI1, AMD1, GOT1 and SRM were tested in Hela-S3 cellsinactivated of NF-YB: GOT 1 was unaffected, ADI1 was substantially decreased, AMD1 and SRM decreased with one of the shRNAs used (Fig. 6B and Figure S11).

AMD1 and ODC1 are deregulated in a number of cancers, notably prostate adenocarcinomas and MYCN-amplified neuroblastomas: in the latter system, ODC1 is required for tumor formation and overexpression predicts patient survival [98]. ODC1 pharmacological inhibition leads to normalization of several pathways -NMYC levels, LIN28/Let-7 expression and glycolysis- altered in neuroblastomas, and it is currently tested in clinical trials [99]. Interestingly, ODC1 and AMD1 are independently involved in mESC self-renewal, in assays of LIF-deprived cells, and can increase reprogramming of differentiated fibroblasts by OCT4, SOX2 and KLF4 [100]. NF-Y is also involved in mESC self-renewal [12, 101, 102]: a positive role on expression of the three TFs was reported, as an important part of the cohort of NF-Y targets involved in stem cells maintenance.

Many genes of the purine pathway are under NF-Y control (Fig. S13); two targets are *bona fide* tumor suppressors: MTAP, frequently deleted in many types of cancers along with the neighboring CDKN2A [103], and Adenylate Kinase 2 AK2 [104]. MTAP shows differential AK2 shows regulation by NF-Y, repressed in cancer cells and in mES cells. As for the synthesis of dNTPs required for the DNA biosynthetic pathways (DNA replication and repair), the key step is the reduction from ribonucleotides to desossiribonucleotides, performed by the rate-limiting enzyme Ribonucleotide Reductase (RnR), an heterotetramer composed of two large (RRM1) and two small (RRM2) subunits. While RRM1 is abundant and constant throughout the cell-cycle, the limiting RRM2 is transcribed only in S-phase [105]. RRM1 is regulated by NF-Y in Hela-S3 cells, but the dependence is higher for RRM2 (Fig. S13), whose promoter contains three functionally crucial CCAAT boxes [106], conserved and important also in Zebrafish [107]. Recently, RRM2 has attracted considerable therapeutic interest, because its targeting leads cancer cells to senesce [108, 109]. It is important to note that cells inactivated of NF-YA are crippled in S-phase progression, develop signs of DNA-damage, with subsequent triggering of an apoptotic response [12, 13]: it is possible that a decrease in the dNTPs pools, due to a decrease in the conversion from NTPs as a consequence of the RRM2 drop, might be at least partially responsible for this behavior.

## CONCLUSIONS AND PERSPECTIVES

**Figure 7:**
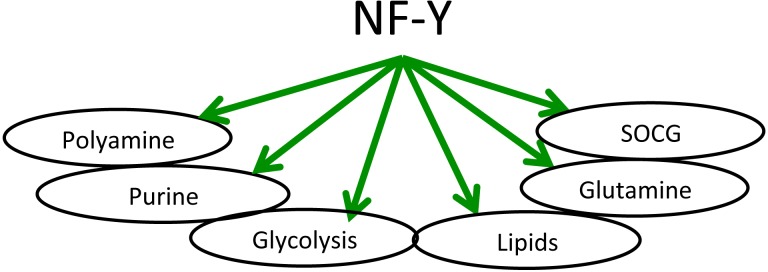
Schematic representation of metabolic pathways regulated by NF-Y

The detailed analysis of metabolic genes under the control of NF-Y described here and summarized in Fig. 7 opens several future avenues of investigations: (i) A thorough phylogenetic analysis of the conservation of CCAAT boxes in metabolic genes targeted by NF-Y should be performed; specifically, genes involved in energy production should be examined, to identify when the switch from aerobic -in yeast- to anaerobic -in mammals- energy production has occurred during evolution: (ii) Further studies should be undertaken on the many NF-Y metabolic targets whose role in cancer development is unknown. Systematic investigation of their expression levels in large cohorts of gene expression data of cancer cells could point at additional regulatory nodes to be further examined experimentally by genetic and biochemical means: (iii) A few TFs known to impact on transcription of metabolic genes emerged, and some have been associated to NF-Y activity; in this regard, the SREBP analysis performed here is illustrative of what can be done, given the availability of large datasets of TFs genomic sites; the identification of major NF-Y partners in the regulation of metabolic genes will shed light on the arrangement of promoter sites and reciprocal interplay. Such catalogue will help our understanding of regulatory circuits of cancer cells.

## MATERIALS AND METHODS

### Lentiviral knockdown and gene expression arrays

Scrambled control (shSC), NF-YA (shNF-YA) and two NF-YB (shNF-YB-1 -gctatgtctactttaggcttt-; shNF-YB-2 -ccaaagaatgtgttcaagaatc-) shRNAs were cloned into pLKO.1 vector (Sigma Aldrich), and viral production and transduction were carried out as previously described [13]. H322 and HCT116 cells were transduced with shSC or shNF-YA viral supernatants, in triplicate, and cells collected after 72 hrs of incubation. Hela-S3 cells were infected with shSC or shNF-YB or shNF-YA and collected at 72 hrs after infection. In the experiments shown in Figure S11, transient DNA transfections with shSC and shNF-YB-2 were performed in triplicate with Lipofectamine 2000 (Invitrogen 11668027). Cells were collected after 72 hours. Knockdown efficiency was assayed by PCR on cDNAs and by Western Blots on whole cell protein extracts using anti-NF-YA, anti NF-YB and anti-Actin antibodies. Total RNA was prepared by Trizol extraction and retrotrascribed with Iscript cDNA Synthesis kit (BIORAD 170-8890). For arrays, RNA was prepared according to Affymetrix standard protocol and hybridized to Hu-Gene 2.0 expression arrays.

### Gene expression analyses

For HeLa-S3 cells, raw data were retrieved from GSE40215 [11]; for mouse embryonic stem cells, raw data were retrieved from GSE56840 [12]. For gene expression analysis of HCT116 and H322 using the Affymetrix platform, biological triplicates of control and shNF-YA-treated cells were independently processed: normalization (rma), quality controlled, probe set filtered, identification of differentially expressed probe sets and annotation of those probe sets to gene symbols were performed using Bioconductor packages (Affy and Limma). We defined upregulated and downregulated genes when the fold change is above 1.3 and FDR <0.05. Gene Ontologies and pathways analyses were performed using the KOBAS 2.0 tool with default settings [20]. Raw data have been deposited in GEO Repository under the accession number GSE70543.

### Immunoblots

For Western Blot analysis, NF-Y-inactivated and control cells were lysed in lysis buffer (50mM Tris–HCl pH 8.0, 120mM NaCl, 1% Triton X100, 20%SDS, 1mM EDTA, protease and phosphatase inhibitors). Equivalent amount of extracts were run on SDS-PAGE, transferred to nitrocellulose membrane (Whatman) and immunoblotted with the following antibodies: anti-Actin (sc-1616, Santa Cruz) anti-NF-YA (Mab1a), anti-NF-YB (Pab001, GeneSpin), anti-Glycolytic enzymes (Glycolysis 2 kit, Cell Signaling 8337S), anti-Vinculin (SAB4200080, Sigma Aldrich).

### qRT-PCR

The *Primer3 0.4.0.* program was used for primers design using default parameters. See Fig. S14 for primer sequences. qPCR was performed with a Biorad myIQ instrument: values are normalized over an internal control ribosomal gene used as normalizator -RPS20- and are represented as fold-enrichment over control sample shSC. Data are presented as mean ± standard error of fold change of 3 biological replicates run in qPCR triplicates. Statistical significance are assessed with one sample t-test and indicated with asterisk when p<0.05.

## SUPPLEMENTARY MATERIAL FIGURES AND TABLES










