# Supplementary material for: NF-Y activates genes of metabolic pathways altered in cancer cells
Source: Oncotarget. 2015 Dec 3;7(2):1633–50. doi: 10.18632/oncotarget.6453 (PMC4811486; doi:10.18632/oncotarget.6453)
Supplement: Supplementary file 1 [file oncotarget-07-1633-s001.pdf]

# NF-Y activates genes of metabolic pathways altered in cancer cells

**Supplementary Material**

Fig.S1 Inactivation of NF-YA in HCT116 and H322 cell lines.

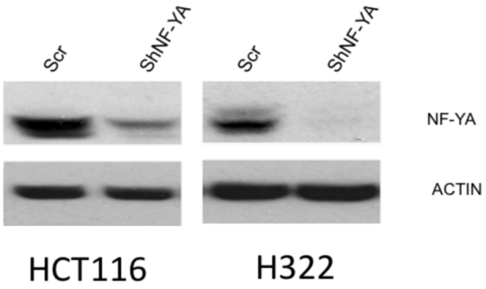


Cells were transduced with shSC or shNF-YA viruses and collected 72 hrs after treatment.

Fig.S3 Global Metabolic maps of genes deregulated after NF-YA inactivation in HCT116 and H322 cells

Genes upregulated after NF-YA silencing — Genes downregulated after NF-YA silencing

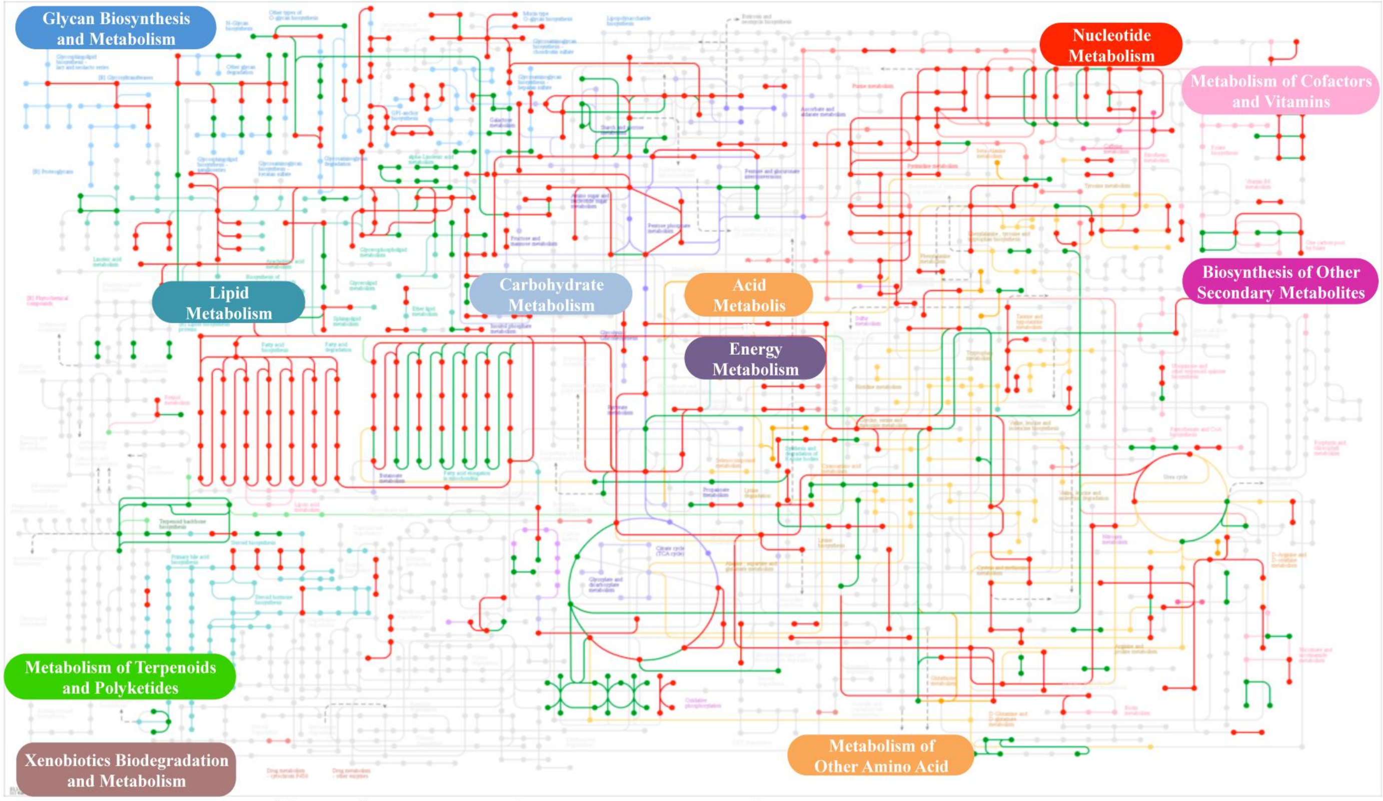


Gene expression profiling after NF-YA silencing in H322 cells

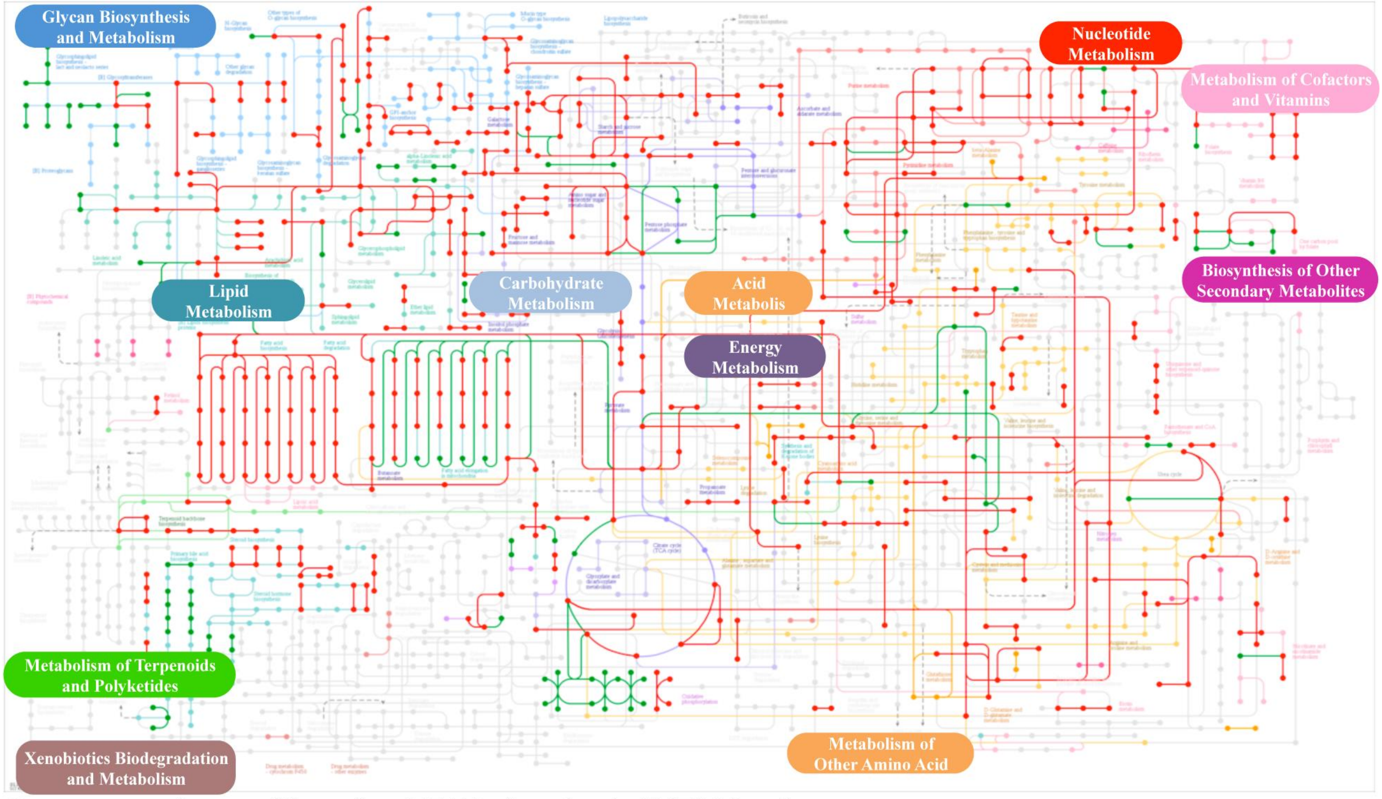


Gene expression profiling after NF-YA silencing in HCT116 cells

Fig.S4 Global Metabolic maps of genes deregulated after NF-YA, NF-YB, NF-YC and NF-Y (all subunits) inactivations in mouse embryonic stem cells.

Genes upregulated after NF-Y silencing — Genes downregulated after NF-Y silencing

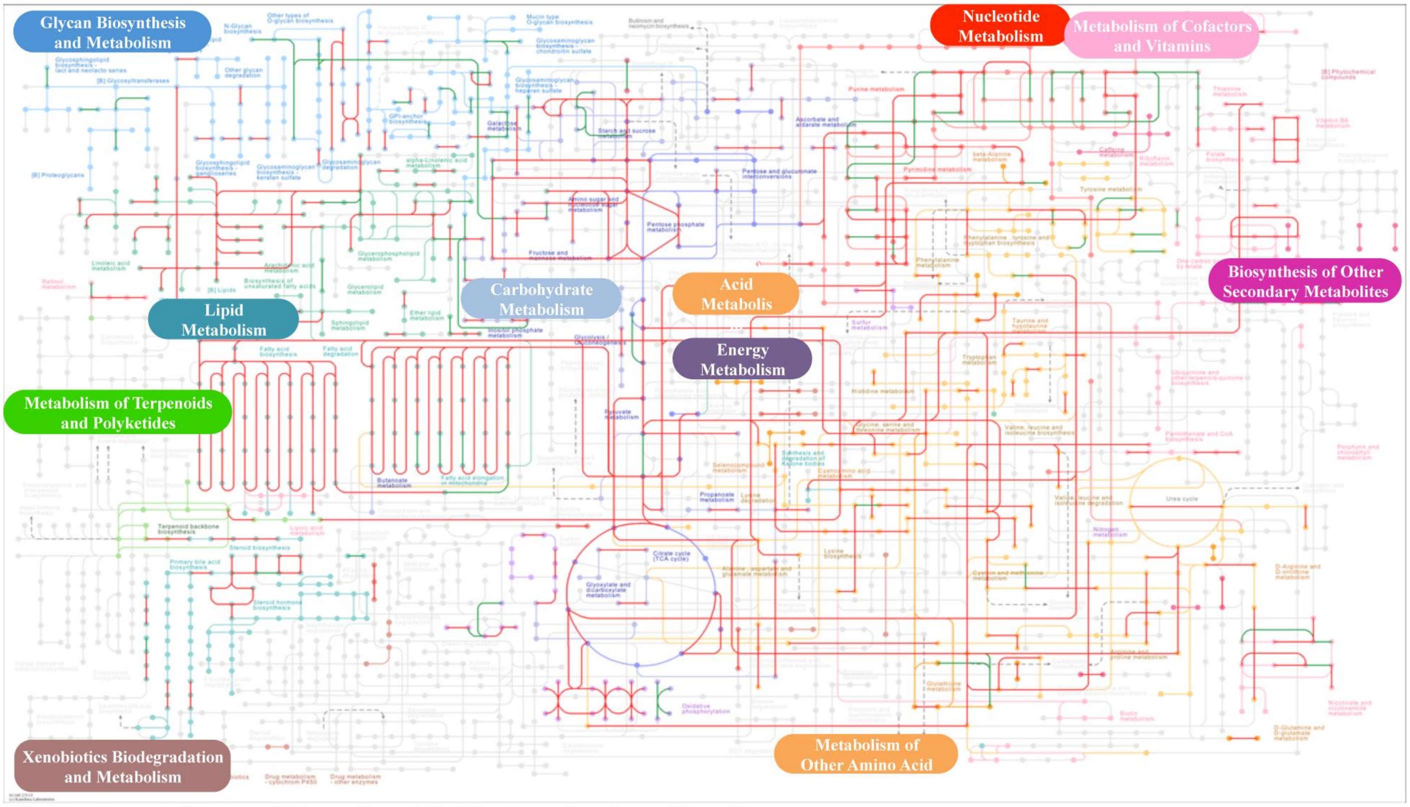


Gene expression profiling after NF-YA silencing in mES cells

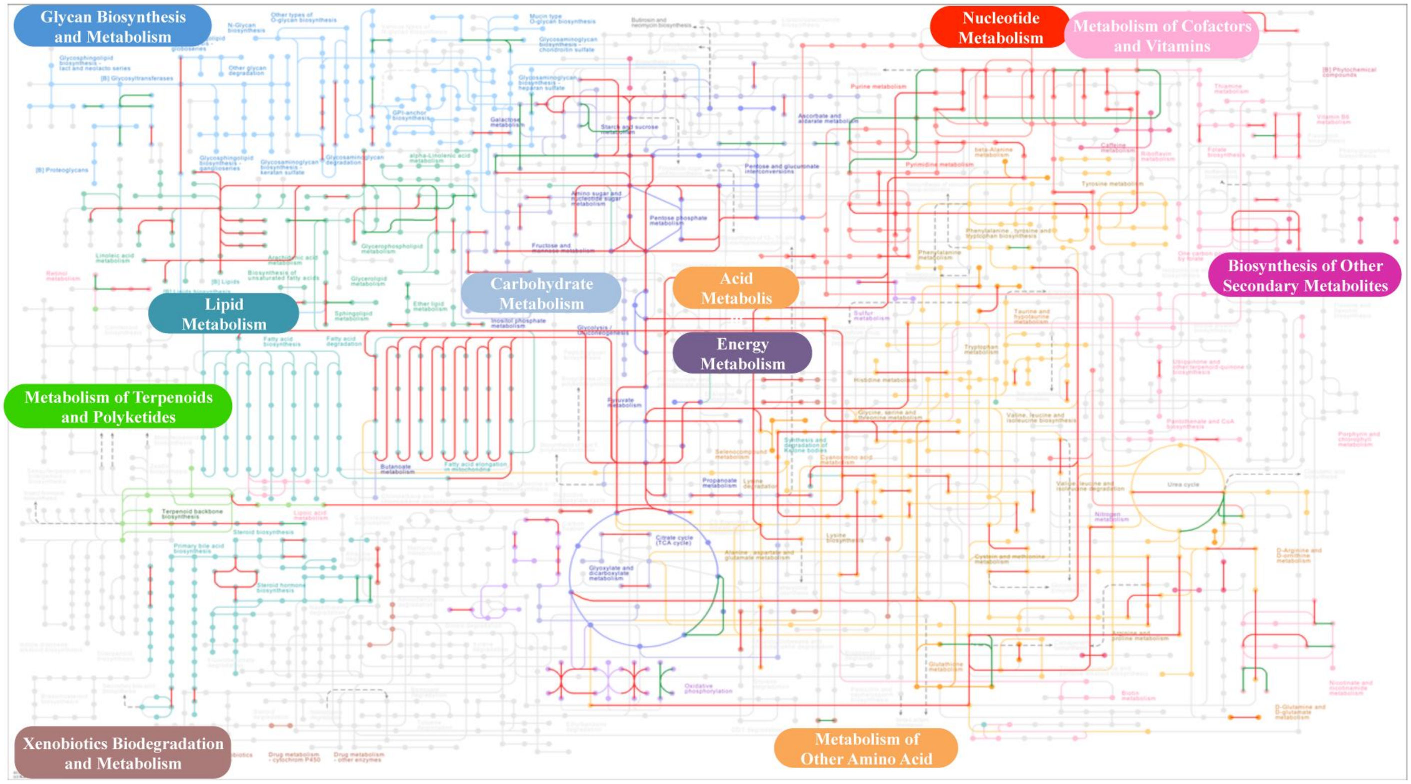


Gene expression profiling after NF-YB silencing in mES cells

Genes upregulated after NF-Y silencing     Genes downregulated after NF-Y silencing

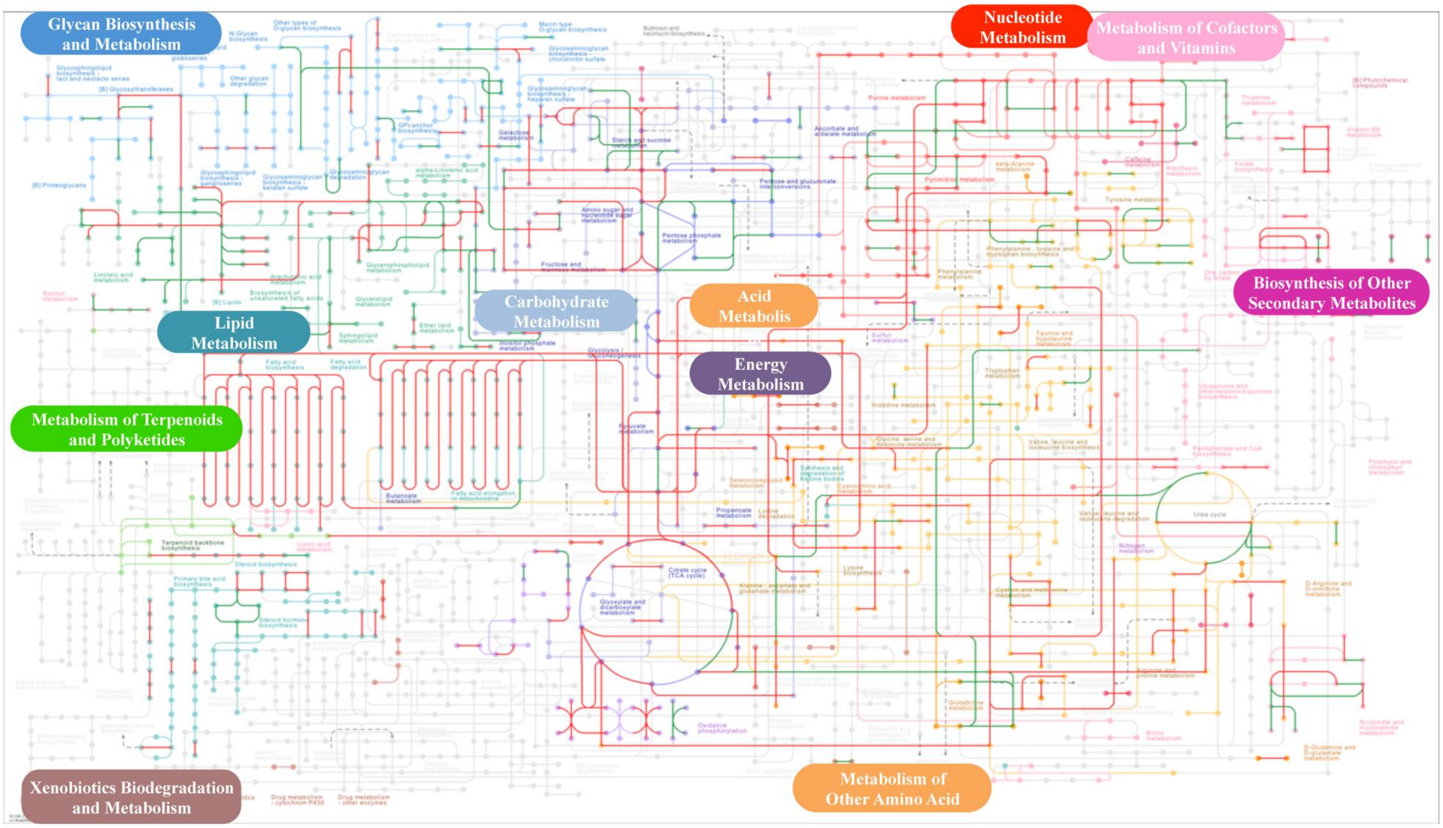


Gene expression profiling after NF-YC silencing in mES cells

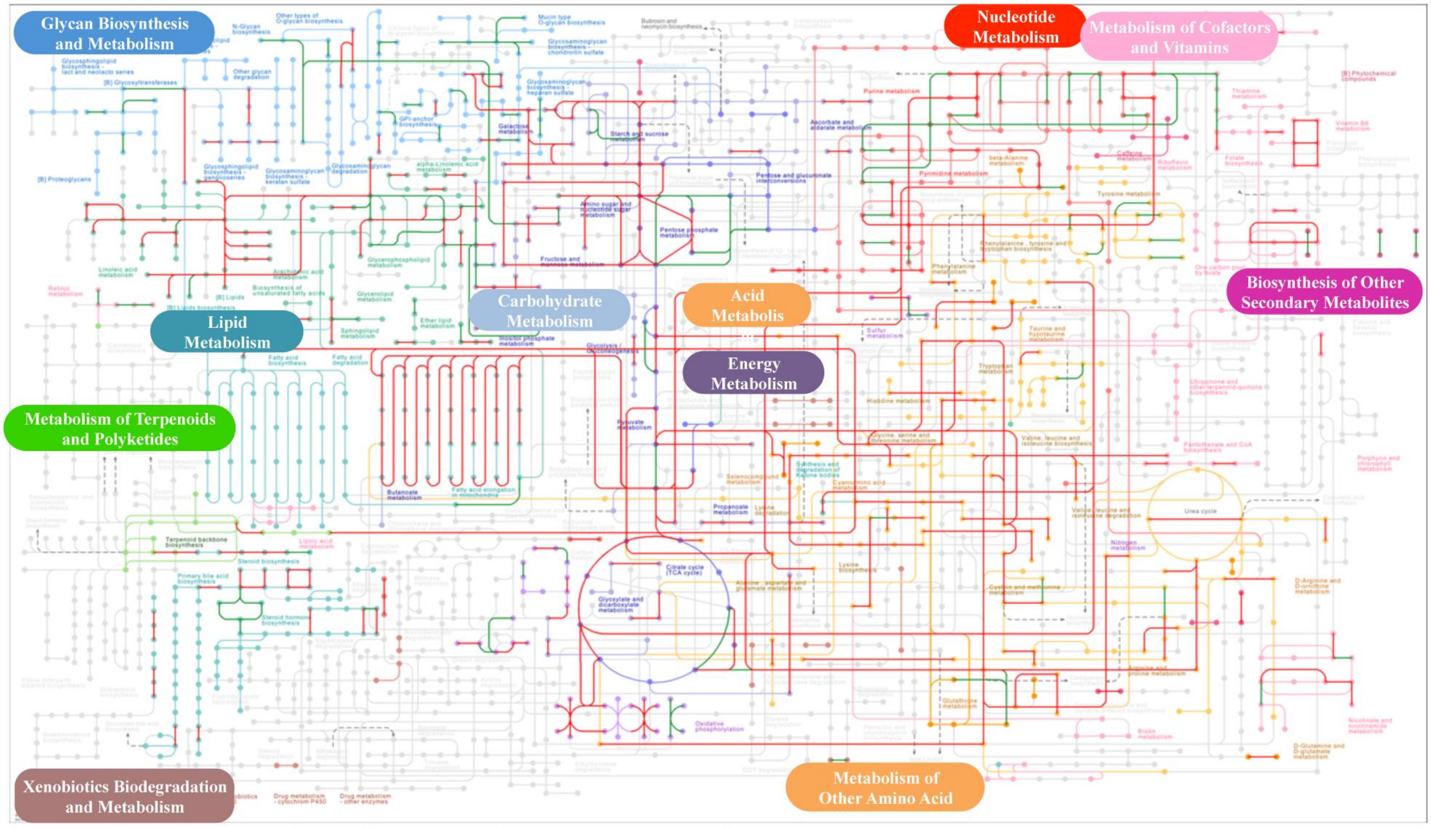


Gene expression profiling after NF-Y silencing in mES cells

Fig.S5 Global Metabolic maps of genes bound in their promoters by NF-YB according to ENCODE data in HeLa cells.

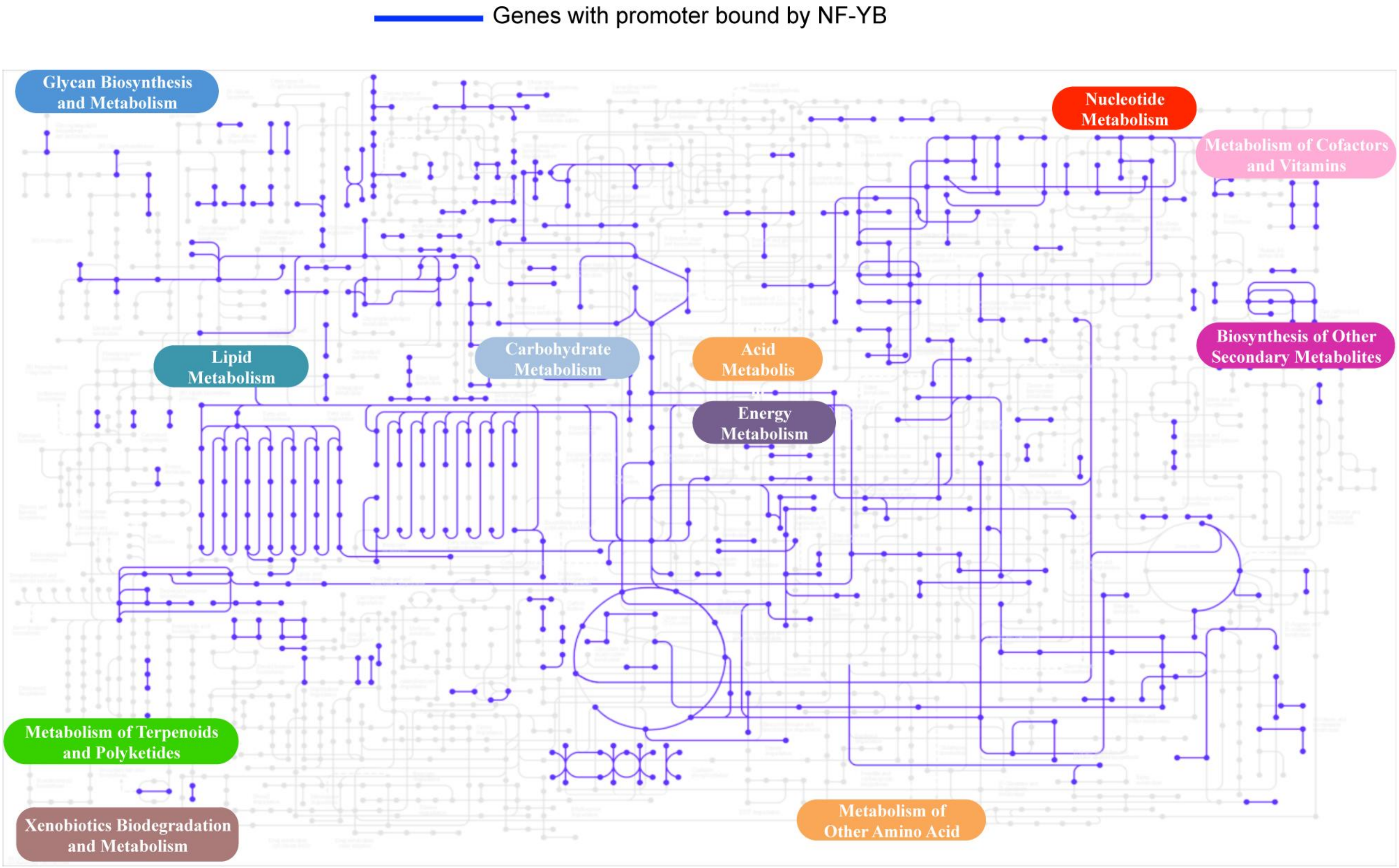


NF-YB binding in Hela cells

Fig.S6 Global Metabolic maps of genes bound in their promoters by NF-YA according to ENCODE data in K562 and GM12878 cells.

Genes with promoter bound by NF-YA

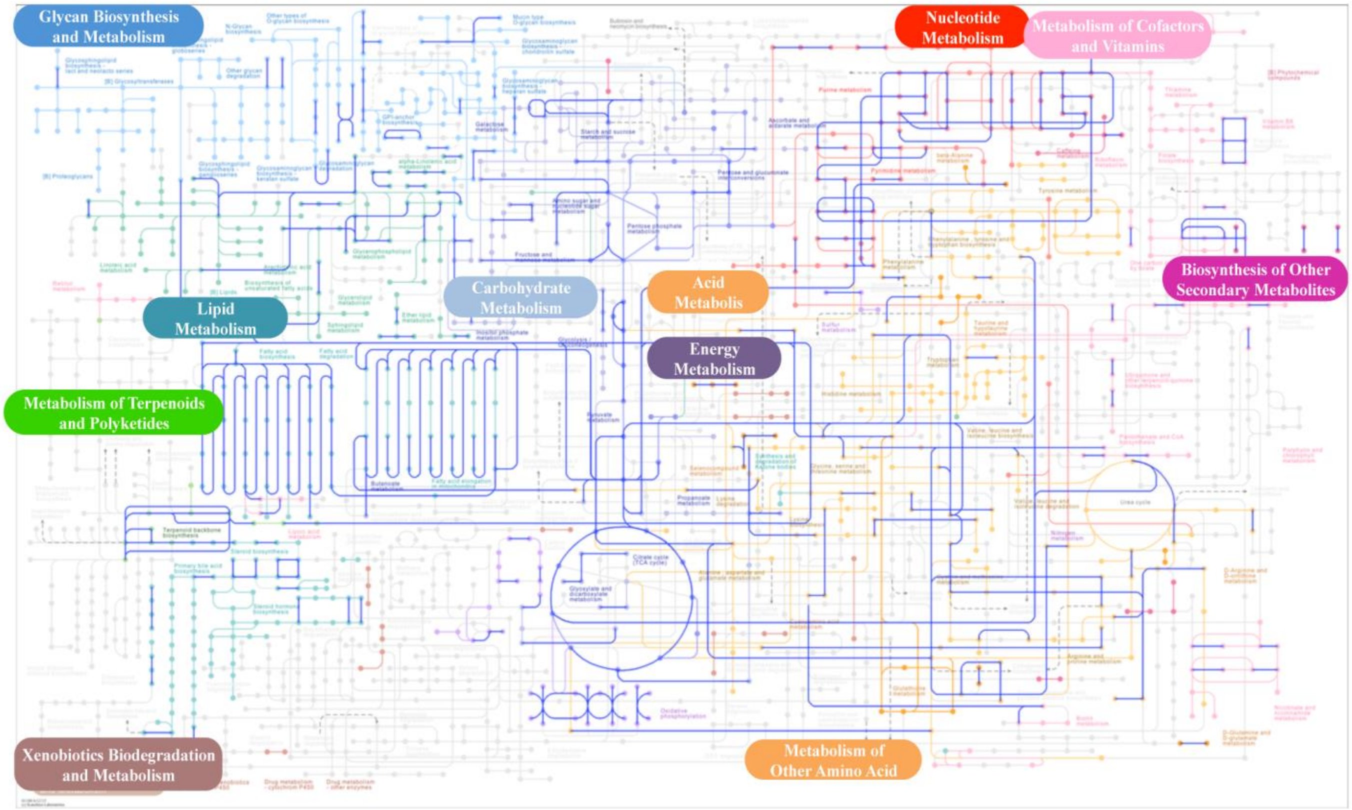


NF-YA binding in K562 cells

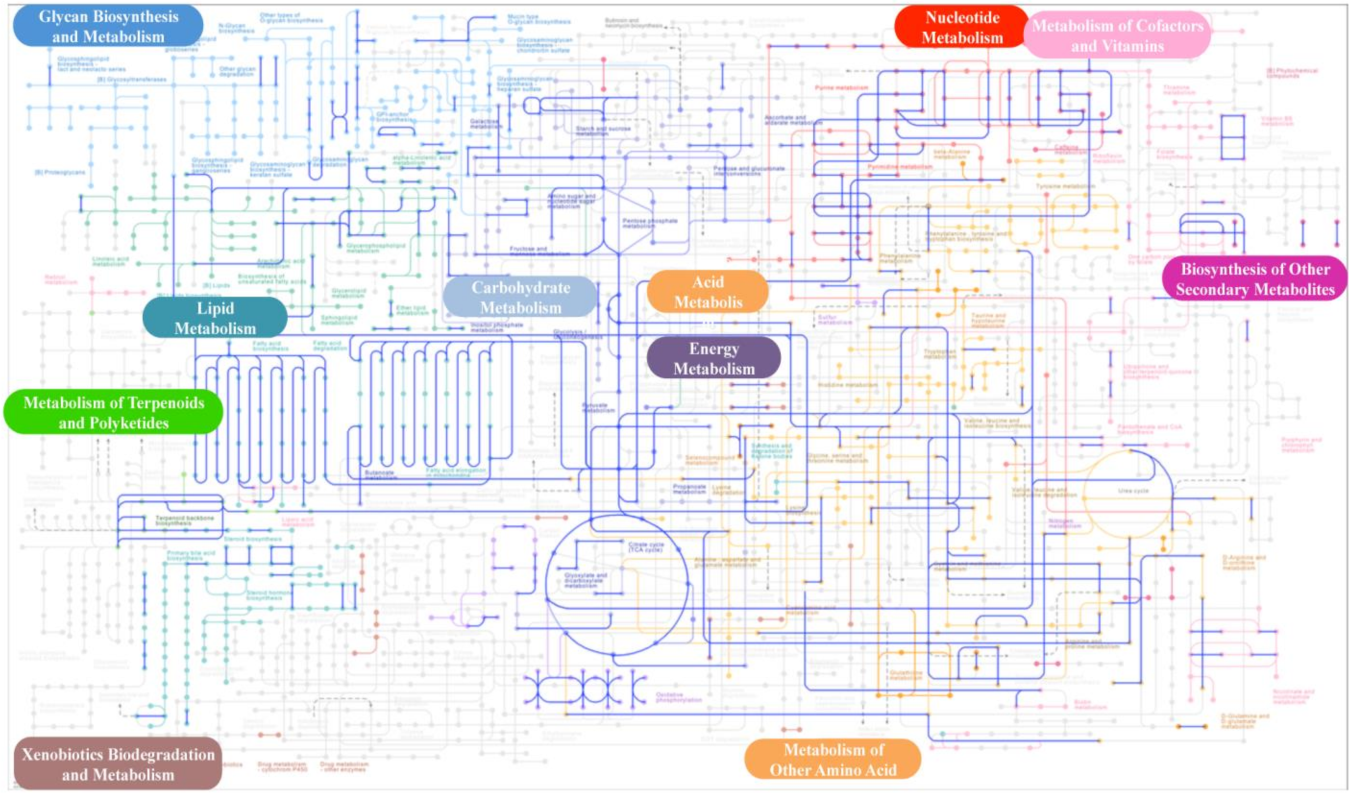


NF-YA binding in GM12878 cells

Fig.S7 Global Metabolic maps of genes bound in their promoters by NF-YB according to ENCODE data in K562 and GM12878 cells.

Genes with promoter bound by NF-YB

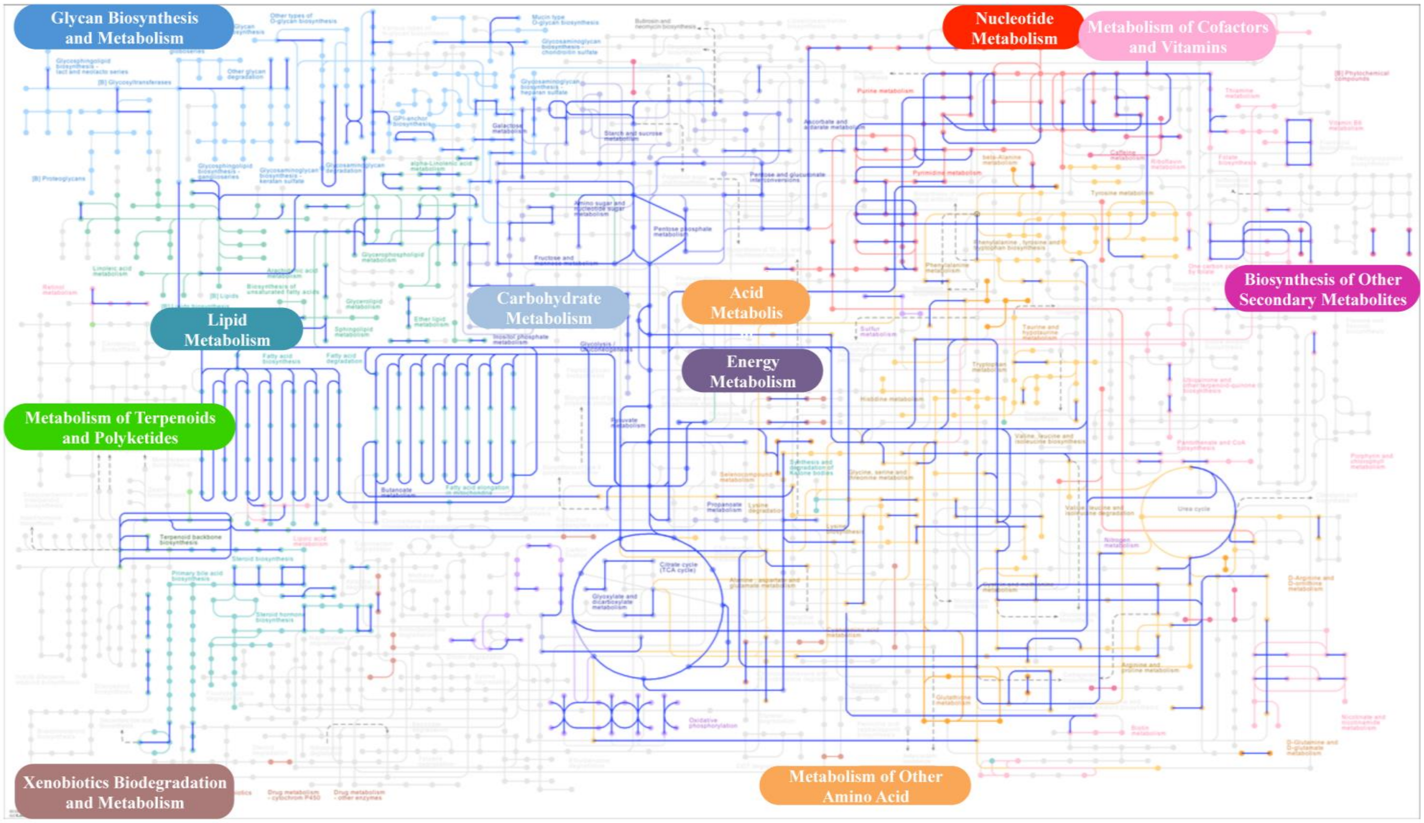


NF-YB binding in K562 cells

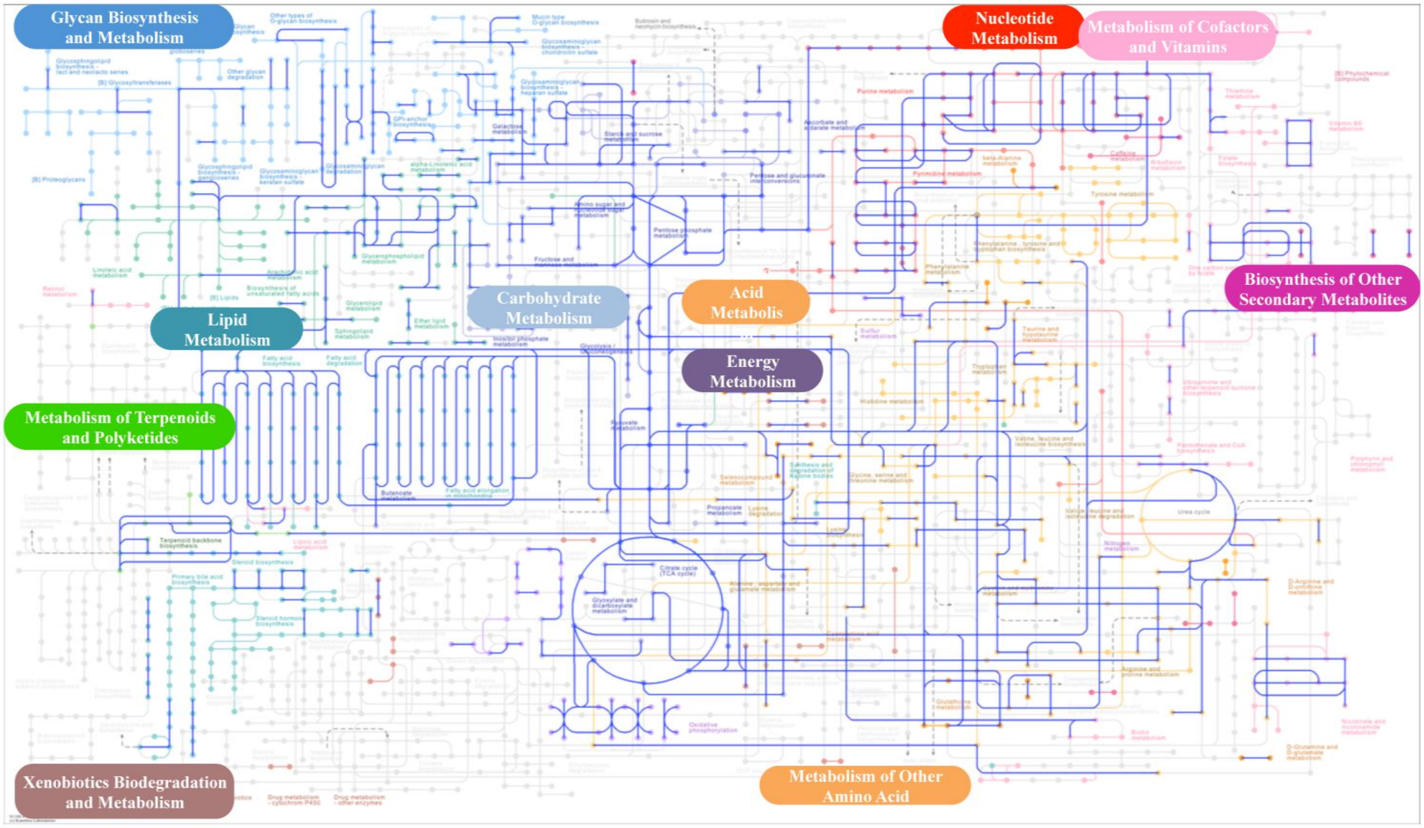


NF-YB binding in GM12878 cells

Fig.S8 Global Metabolic maps of genes bound in their promoters by NF-YA, NF-YB and NF-YC according to data published by Oldfield AJ et al..

Genes with promoter bound by NF-Y

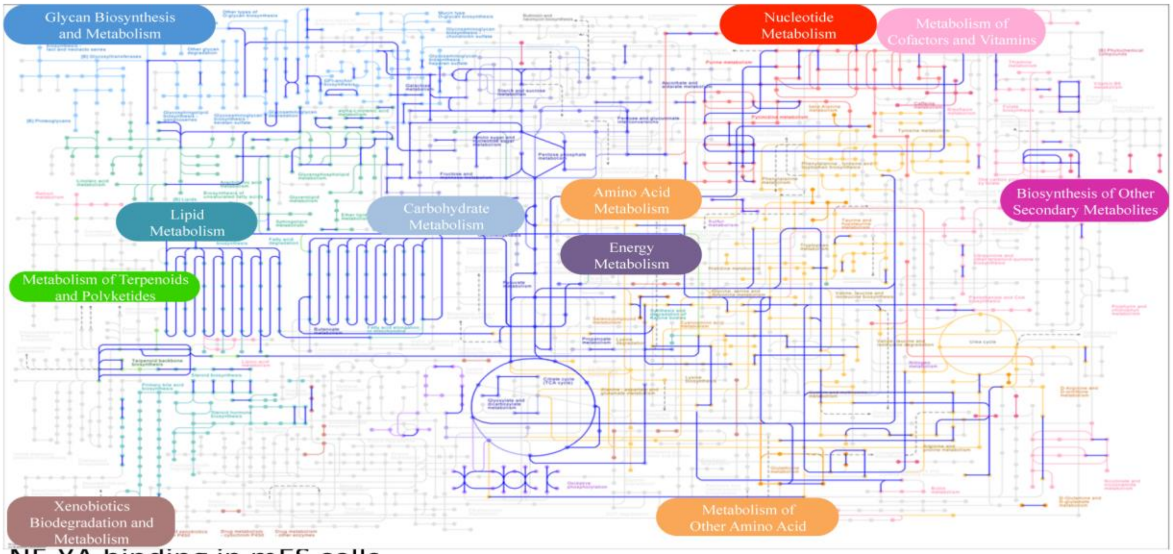


NF-YA binding in mES cells

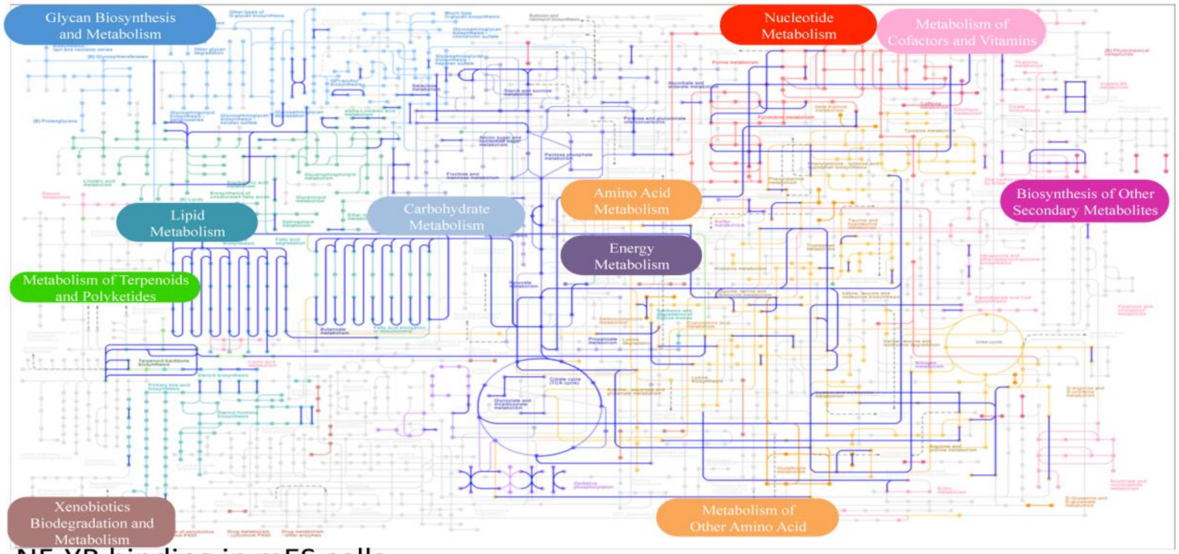


NF-YB binding in mES cells

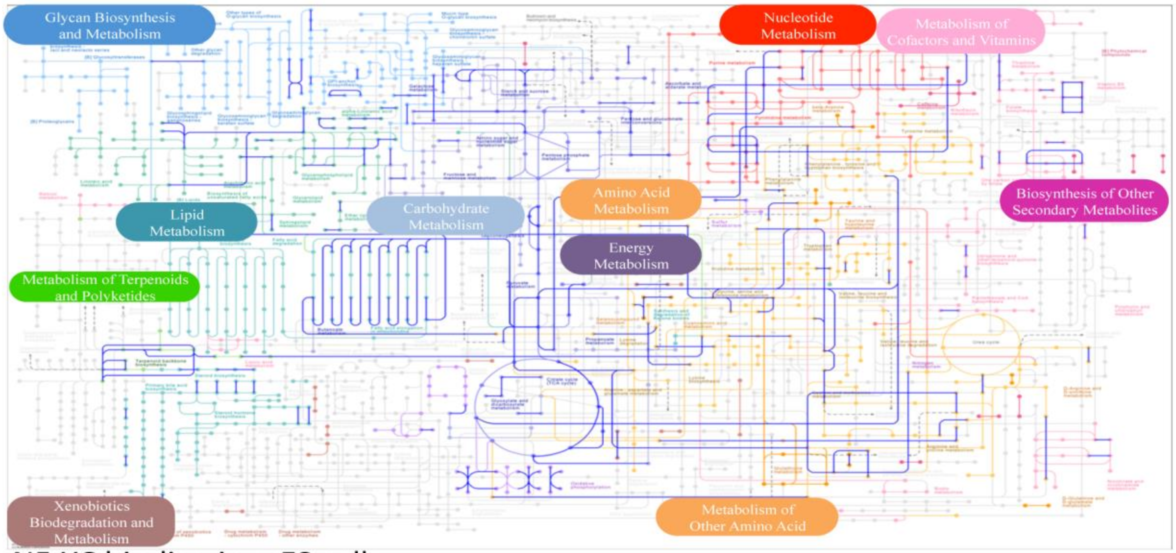


NF-YC binding in mES cells

Fig.S11 Inactivation of NF-YB in Hela cells using sh-NF-YB-2 and analysis of expression of targets genes.

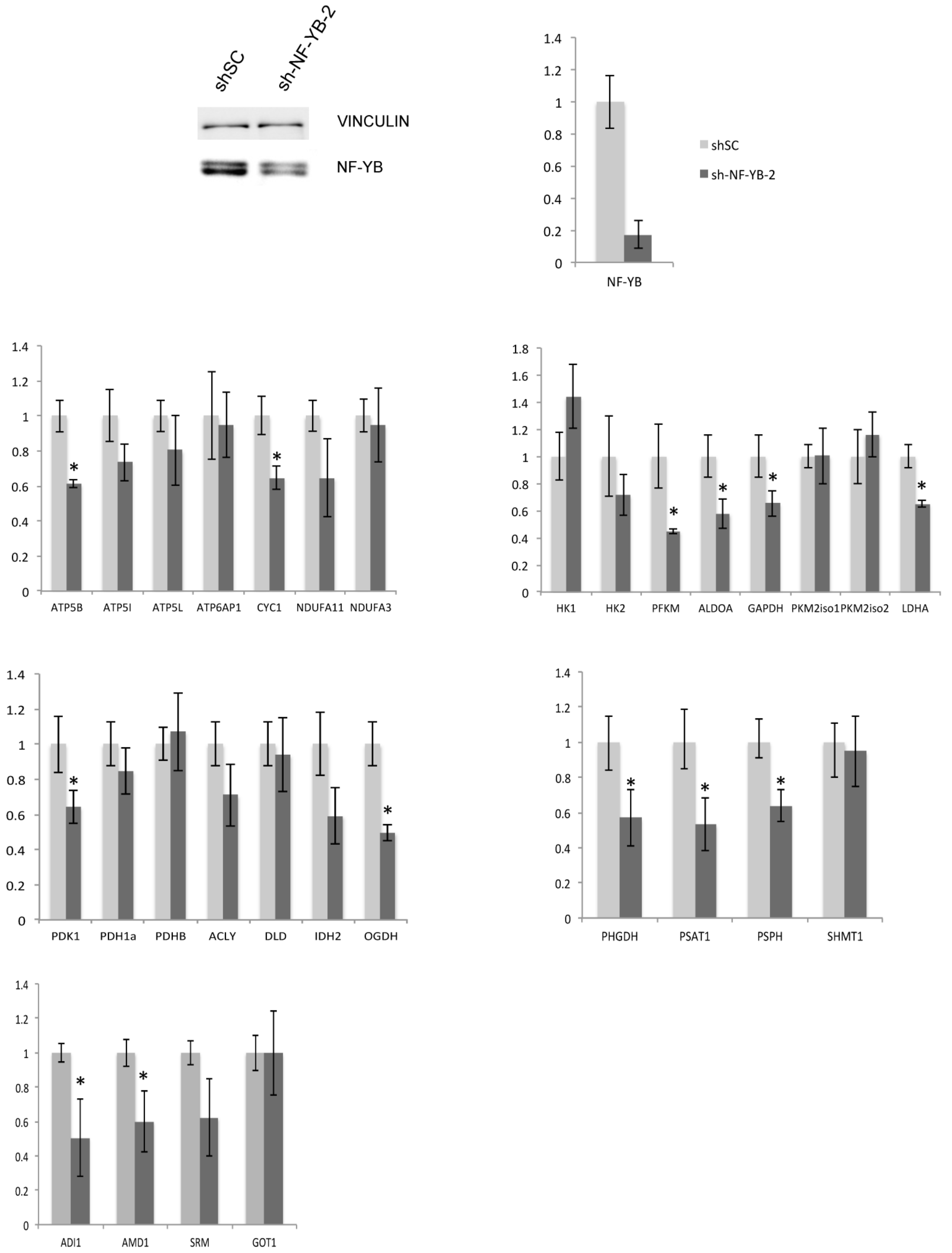


Western blot analysis of protein level of NF-YB in Hela cells inactivated of NF-YB using sh-NF-YB-2. qRT-PCR evaluation of expression levels of NF-YB and selected genes after inactivation of NF-YB in Hela cells. The average -/+ SD of three biological replicates is represented (*p<0.05).

Fig.S12 Regulation of genes of TCA cycle.

A

| | H322 | HCT116 | HeLa-S3 | HELA | | K562 | | GM12787 | | mESc | | | | | | |
|---|---|---|---|---|---|---|---|---|---|---|---|---|---|---|---|---|
| | shYA | shYA | shYA | YA | YB | YA | YB | YA | YB | YA | YB | YC | siYA | siYB | siYC | siNFY |
| PDK1 | | | | | | P | P | | | E | E | E | | | | |
| PDK2 | | | | P | P | P | P | P | P | P | P | P | | | | |
| PDK3 | | | | P | P | | P | | P | | | | | | | |
| PDK4 | | | | P | P | | P | | P | P | P | P | | | | |
| PDHA1 | | | | P | P | P | P | P | P | P | | | | | | |
| PDHA2 | | | | | | | | | | | | | | | | |
| PDHB | | | | P | | | | | P | P | P | P | | | | |
| DLAT | | | | | | | | | P | | | | | | | |
| PCK1 | | | | | | | | | | | | | | | | |
| PCK2 | | | | P | PEi | Ei | PEi | Ei | PEi | P | | | | | | |
| PC | | | | PE | PE | PE | PE | PE | PE | P | P | P | | | | |
| ACLY | | | | P | P | P | P | P | P | P | P | | | | | |
| CS | | | | P | P | P | P | P | P | P | P | | | | | |
| ACO1 | | | | | | | | | | | | | | | | |
| ACO2 | | | | P | P | P | P | P | P | | | | | | | |
| IDH1 | | | | | | | | | | | | | | | | |
| IDH2 | | | | PEi | Ei | Ei | PEi | | PEi | | | | | | | |
| IDH3A | | | | P | P | | P | | P | | | | | | | |
| IDH3B | | | | | | | | | P | | | | | | | |
| IDH3G | | | | P | P | P | P | P | P | P | P | | | | | |
| OGDH | | | | P | P | P | P | P | P | P | P | P | | | | |
| DLD | | | | P | P | PE | PE | P | PE | P | | | | | | |
| DLST | | | | | | | | | | | | | | | | |
| SUCLA2 | | | | | | | | | | | | | | | | |
| SUCLG1 | | | | | P | | P | | P | | | | | | | |
| SUCLG2 | | | | P | P | P | P | P | P | | | | | | | |
| SDHA | | | | | | | | | | | | | | | | |
| SDHB | | | | P | | | | | P | | | | | | | |
| SDHC | | | | | | | | | | | | | | | | |
| SDHD | | | | | | | | | | | | | | | | |
| FH | | | | P | P | P | P | P | P | P | P | | | | | |
| MDH1 | | | | P | P | P | P | P | P | P | | | | | | |
| MDH2 | | | | | | | | | | | | | | | | |

-1.5 log2FC +1.5

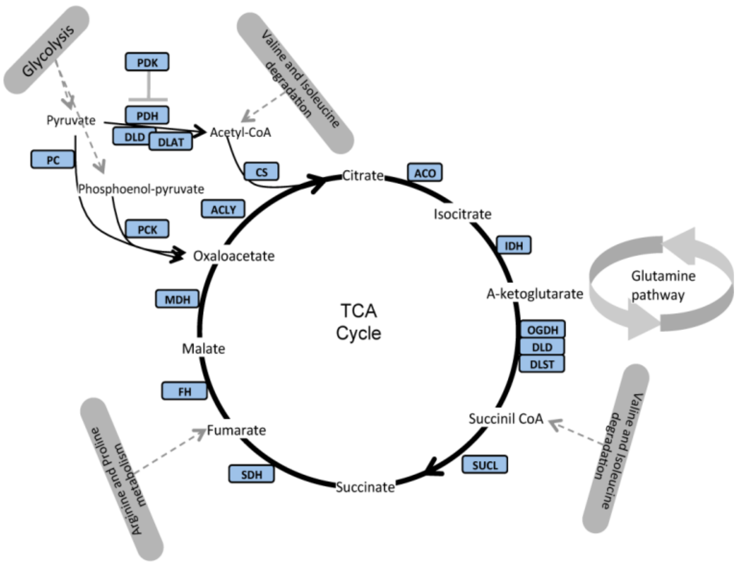


B

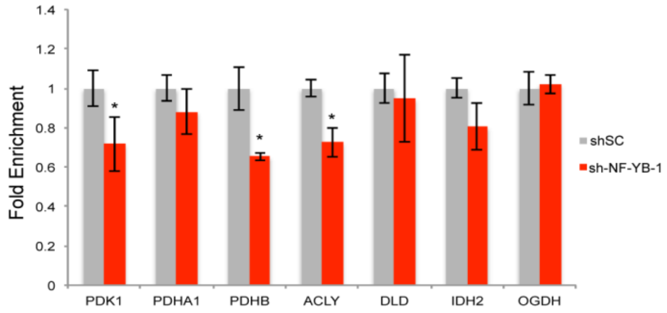


C

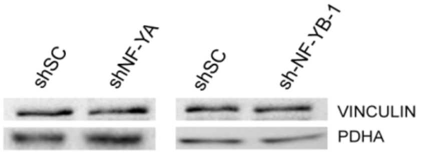


**A**) Expression levels of genes encoding TCA enzymes are shown after inactivation of NF-Y in different cell lines, and the presence of NF-Y binding is indicated as in Fig. 3. **B**) qRT-PCR validation of expression levels of selected genes after inactivation of NF-YB in Hela cells. The average -/+ SD of three biological replicates is represented (*p<0.05). **C**) Western blot analysis of protein levels of PDHA1 in Hela cells inactivated of NF-YA (Left panel) or NF-YB (Right panel).

FigS13 Activation of genes of purine and pyrimidine metabolisms by NF-Y.

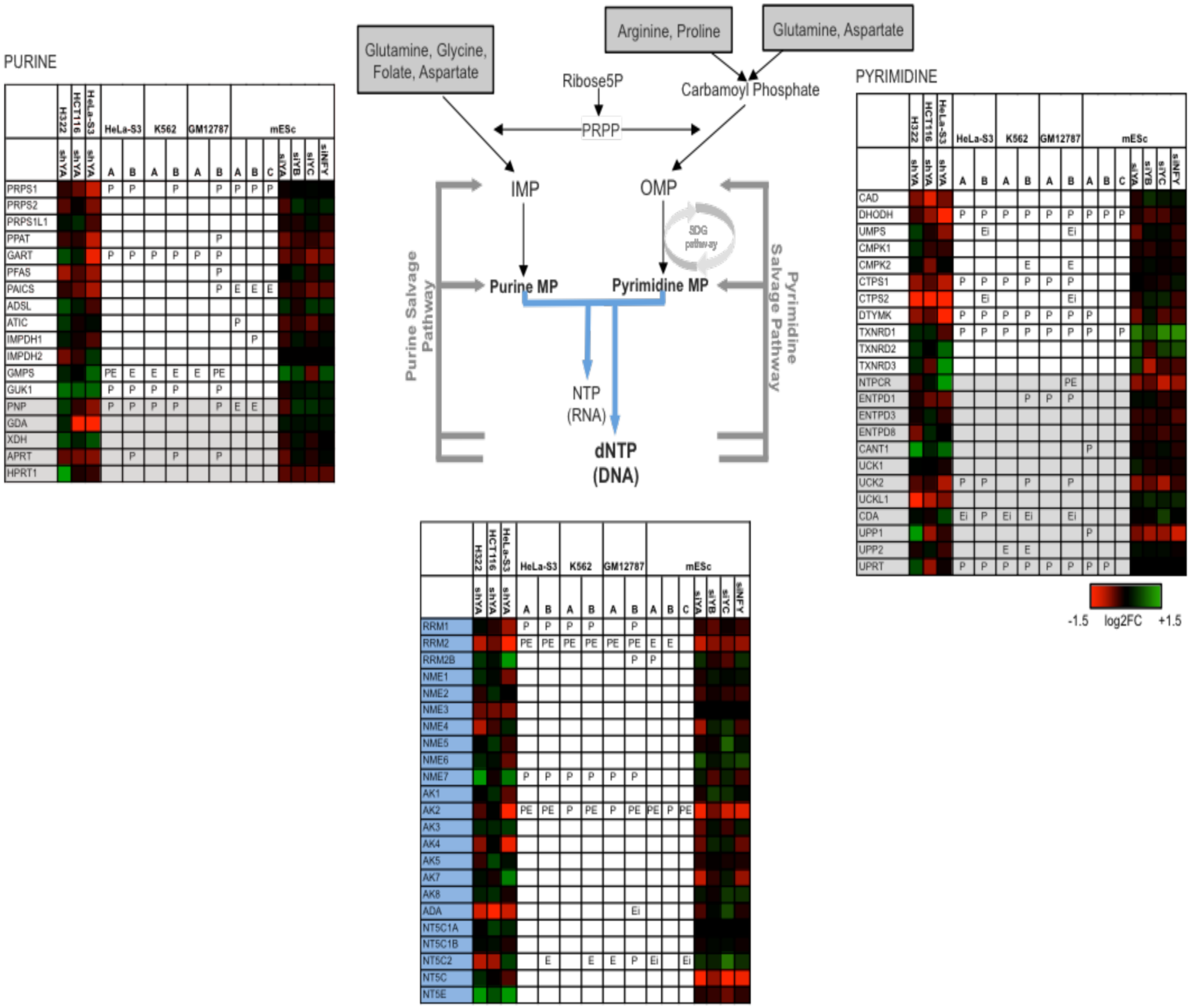


Genes of the purine and pyrimidine metabolisms are shown with the expression levels after inactivation of NF-Y, and the presence of *in vivo* NF-Y binding.
